# Single-cell analysis of CD4+ T-cell differentiation reveals three major cell states and progressive acceleration of proliferation

**DOI:** 10.1186/s13059-016-0957-5

**Published:** 2016-05-12

**Authors:** Valentina Proserpio, Andrea Piccolo, Liora Haim-Vilmovsky, Gozde Kar, Tapio Lönnberg, Valentine Svensson, Jhuma Pramanik, Kedar Nath Natarajan, Weichao Zhai, Xiuwei Zhang, Giacomo Donati, Melis Kayikci, Jurij Kotar, Andrew N. J. McKenzie, Ruddy Montandon, Oliver Billker, Steven Woodhouse, Pietro Cicuta, Mario Nicodemi, Sarah A. Teichmann

**Affiliations:** EMBL, European Bioinformatics Institute (EBI), Hinxton, CB10 1SD UK; Department of Physics, University of Naples Federico II, CNR-Spin, Istituto Nazionale di Fisica Nucleare (INFN), Napoli, Italy; Wellcome Trust Sanger Institute, Wellcome Trust Genome Campus, Hinxton, Cambridge CB10 1SA UK; Cavendish Laboratory, University of Cambridge, Madingley Road, Cambridge, CB3 0HE UK; Centre for Stem Cells and Regenerative Medicine, Kings College London, London, SE1 9RT UK; MRC Laboratory of Molecular Biology, Cambridge, CB2 0QH UK; Department of Haematology, Cambridge Institute for Medical Research, University of Cambridge, Cambridge Biomedical Campus, Wellcome Trust/MRC Building, Hills Road, Cambridge, CB2 0XY UK; Wellcome Trust—Medical Research Council Cambridge Stem Cell Institute, University of Cambridge, Cambridge, UK

**Keywords:** CD4+ T cells, Adaptive immunity, Single-cell RNA-seq, Cell cycle, Differentiation, Live imaging

## Abstract

**Background:**

Differentiation of lymphocytes is frequently accompanied by cell cycle changes, interplay that is of central importance for immunity but is still incompletely understood. Here, we interrogate and quantitatively model how proliferation is linked to differentiation in CD4+ T cells.

**Results:**

We perform ex vivo single-cell RNA-sequencing of CD4+ T cells during a mouse model of infection that elicits a type 2 immune response and infer that the differentiated, cytokine-producing cells cycle faster than early activated precursor cells. To dissect this phenomenon quantitatively, we determine expression profiles across consecutive generations of differentiated and undifferentiated cells during Th2 polarization in vitro. We predict three discrete cell states, which we verify by single-cell quantitative PCR. Based on these three states, we extract rates of death, division and differentiation with a branching state Markov model to describe the cell population dynamics. From this multi-scale modelling, we infer a significant acceleration in proliferation from the intermediate activated cell state to the mature cytokine-secreting effector state. We confirm this acceleration both by live imaging of single Th2 cells and in an ex vivo Th1 malaria model by single-cell RNA-sequencing.

**Conclusion:**

The link between cytokine secretion and proliferation rate holds both in Th1 and Th2 cells in vivo and in vitro, indicating that this is likely a general phenomenon in adaptive immunity.

**Electronic supplementary material:**

The online version of this article (doi:10.1186/s13059-016-0957-5) contains supplementary material, which is available to authorized users.

## Background

Many differentiation processes occur hand-in-hand with a change in cell cycle status: this can be cell cycle arrest, as in the monocyte to macrophage transition [1], cell cycle entry, as for the pre-adipocyte to adipocyte differentiation [2], and entry and subsequent cell division, as in T helper (Th) cell differentiation [3]. Th cell differentiation is the process where naïve CD4+ T cells transition to effector lymphocytes and is central to mammalian adaptive immunity. After antigen stimulation of the T-cell receptor in the presence of specific cytokines, naïve Th cells start dividing rapidly to reach a differentiated state, with the best understood being Th1, Th2, Th17 and pTregs [4]. So far, several master regulators have been identified (e.g. *Gata3* for Th2, *T-bet* for Th1, *Rorgt* for Th17 and *Foxp3* for pTregs) [4] and there is considerable insight into their regulatory networks [5]. While much is known in CD8+ (killer) T cells [6], the expansion of CD4+ (helper) T cells during an infection is less well understood at the cellular and molecular levels.

How does the coupling between differentiation and the cell cycle occur in CD4+ T cells? Are the two processes independent and orthogonal, as suggested by Duffy and Hodgkin [7], or linked through molecules and hence intertwined [8]? Does differentiation occur in a gradual manner as suggested by many studies, including a recent single-cell analysis of lung epithelial development [9], or in a cooperative switch-like manner?

Here, we use a new approach to tackle these questions, which is to extract biologically intermediate states of differentiation from a single chronological time point. By sorting out separate cell populations from a single cell culture of asynchronized, dividing cells, we aimed to reduce the biological variability in cytokine exposure, confluence, etc. With this approach, we minimize the biological noise in our data and focus entirely on the processes of cell division and differentiation.

We used in-depth transcriptome profiling coupled with bioinformatics data analysis to identify three major cell states during Th2 differentiation. By counting cells in each cell generation using flow cytometry, we modelled the rates of death, division and differentiation using a discrete time Markov branching process. This revealed a higher cell division rate for differentiated cells compared with proliferating, activated cells. We validate those finding by DNA staining and by single-cell live imaging of Th2 cells. These in vitro data supported the idea of a fine-tuned relationship between cell cycle speed and differentiation status in CD4+ T cells.

Finally, we related our findings from an ex vivo cell culture model of Th2 differentiation to single-cell transcriptomes of Th1 cells from a mouse model of malaria infection. The in vivo cytokine secreting Th1 cells also cycle more quickly than in vivo activated cells, showing the universal relevance of our results to primary activation of T cells. This implies that an acceleration of effector CD4+ T cell expansion upon differentiation is part of the immune system’s mechanism of pathogen clearance during primary activation.

## Results

### Cell division-linked differentiation of Th2 cells in vivo and in vitro

**Fig. 1:**
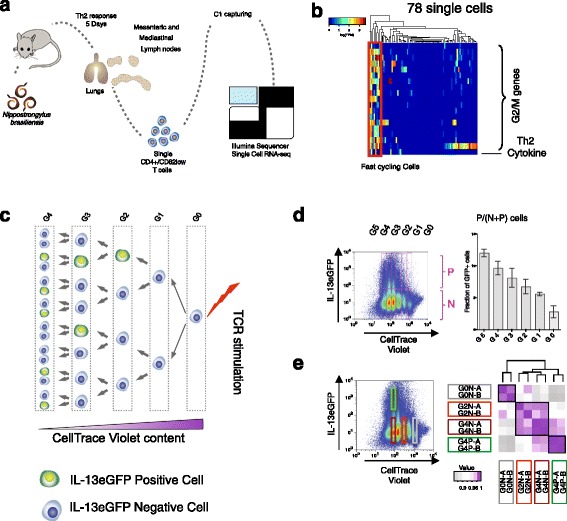
Th2 differentiation resolved by cell generation. **a** Overview of the experiment. CD3+/CD4+/CD62L- T helper cells were isolated from lungs, mesenteric and mediastinal lymph nodes of Nb-infected mice on day 5 post-infection. After cell capturing and cDNA generation with the C1 system, samples were sequenced with an Illumina Hi-seq Sequencer. **b** Seventy-eight single cells were clustered according to the expression of G2/M genes (logTPM) as a measure of cell cycle speed. *TPM* transcripts per millions. Three cells expressing IL4 clustered within the group of cells expressing high levels of G2/M genes (in the red box) (*p* value = 0.008, Fisher’s exact test). **c** Schematic representation of the division/differentiation process from a naïve cell to fully differentiated Th2 cells. The CellTrace Violet content is roughly equally distributed between daughter cells after each mitotic division. Cells expressing the *Il13-eGFP* Th2 differentiation marker are shown in *green. TCR* T-cell receptor. **d** Flow cytometry plot of CellTrace Violet versus *Il13-eGFP* differentiated Th2 cells at day 3.5. Consecutive generations (from G0 to G5) are visualized as *pink gates*. The *upper gates* are IL13-positive cells (*P*), and the *lower gates* contain IL13-negative cells (*N*). Ratio of GFP-P cells to the total number of cells per generation (average and standard deviation of three biological replicates). **e** Cells in the gates highlighted were sorted by FACS and profiled by mRNA-sequencing. Hierarchical clustering of the distance matrices between RNA expression profiles

After antigen stimulation of the T-cell receptor [10], naïve CD4+ T cells start dividing quickly and some cells initiate expression of specific cytokines, which is the hallmark of differentiated effector cells. To probe this process in vivo, we isolated and sequenced CD3+/CD4+/CD62L- single cells from spleen and both mediastinal and mesenteric lymph nodes of *Nippostrongylus brasiliensis* (Nb)-infected mice 5 days post-infection (Fig. 1a). We performed quality control analysis in order to remove cells with a poor quality library (see the “Methods” section for details and Additional file 1: Figure S1a) and we retained data from 78 cells. All read statistics are reported in Additional file 2: Table S1. In order to separate the fast cycling cells from the slow cycling ones, we clustered them according to the expression of cell cycle genes (Fig. 1b). We ranked the cells according to the expression of aggregated G2/M genes as a measure of “cell cycle score”, thus reflecting the speed of the cell cycle (cell cycle gene list is provided in Additional file 3). We observed that the cells expressing higher amounts of G2/M genes were also significantly enriched in interleukin (IL)4 (*p* value = 0.008, Fisher’s exact test). In order to verify that those G2/M high cells were proliferating faster and were enriched in IL4 expression, we looked at the expression level of proliferation marker genes (from previous work [11, 12]). The cells enriched in those genes also expressed significantly higher amounts of IL4 (*p* value = 0.001, Fisher’s exact test), confirming that cytokine-producing cells are cycling faster (Additional file 1: Figure S1b). We excluded that this observation was due to higher library quality of those cells as visualized in Additional file 1: Figure S1c–e. Based on this observation, we proceeded to study the link between cell cycle speed and differentiation in Th2 cells in more details in an in vitro cell culture system.

As a marker of differentiation we employed IL13 instead of IL4, as its expression is less susceptible to changes in IL4 concentration in the medium. Staining precursor naïve cells with CellTrace Violet dye allowed us to discriminate cells that have undergone different numbers of cell divisions (Fig. 1c). Primary cells derived from *Il13-eGFP* homozygous reporter mice allowed us to identify differentiated Th2 cells [11]. Using this system, we observe, consistent with previously published data for other cytokines [8, 12], that the proportion of differentiated cells (with fluorescent IL13+ reporter expression) increases linearly in each consecutive generation (Fig. 1d). In previous reports, cytokine-producing cells have been detected only from the third generation onwards [8], while we detect these cells in earlier generations already. This is probably due to our use of a green fluorescent protein (GFP) reporter for the endogenous cytokine, instead of the traditional staining with fluorescent antibody (see Additional file 1: Figure S1f for traditional antibody staining).

To dissect whether sudden or gradual changes in cell state occur during Th2 differentiation, we performed a transcriptome-wide characterization of cells that had undergone different numbers of mitotic divisions after 3.5 days of activation. From this single time point, we sorted and carried out mRNA-sequencing (mRNA-seq) of three non-overlapping populations of cells that were not expressing GFP and had, respectively, not divided (generation 0 negative (G0N)), divided twice (G2N) or divided four times (G4N). We also profiled a fourth population of cells that had divided four times and was positive for GFP (G4P; Fig. 1e; Additional file 2: Tables S2 and S3).

Hierarchical clustering of these datasets indicates that there are three major transcriptomic states (Fig. 1e). A G0N cluster is clearly separate from the other populations, indicating a major difference between cells that have not yet undergone mitosis and the other cells that have entered the cell cycle. The G4P cluster is more distant from the other two dividing populations, indicating that the expression of one single marker of differentiation (*Il13*) occurs concomitantly with global changes to the expression profile of growing lymphocytes. In contrast, the G2N and G4N data sets cluster together, sharing similar expression profiles (Additional file 1: Figure S1g). Quantitative PCR (qPCR) and flow cytometry of individual genes and proteins supported and validated these conclusions from the mRNA-seq data (Additional file 1: Figure S1h).

### RNA-seq analysis indicates three major cell states

**Fig. 2:**
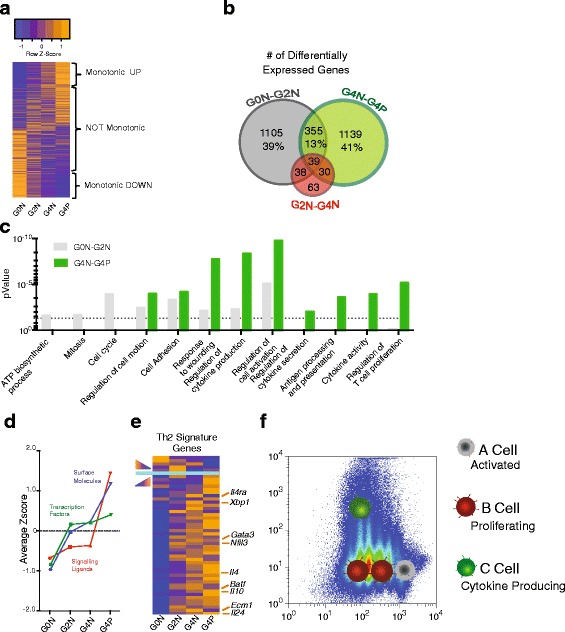
Deep transcriptomic analysis reveals three discrete cell states during Th2 differentiation. **a** Heatmap of all ~14,000 protein-coding genes (*rows*) per generation (*columns*). At the *top* and *bottom*, genes with a monotonic increase/decrease are shown. In the *middle*, genes are ranked according to distance from G4P and G0N. **b** Number and percentage of differentially expressed genes between samples. **c** Gene Ontology enrichment analysis was performed on differentially expressed genes between G0N and G2N (*gray bars*) and G4N and G4P (*green bars*). The threshold *p* value of 0.05 is shown as a *dotted line*. **d** Average Z-scores for upregulated genes belonging to different functional categories calculated from heatmaps in Additional file 4: Figure S2d. **e** Heatmap of Th2 signature gene expression across generations. **f** A three-state differentiation model in which G0N cells are named *A cells* (“*Activated*” cells), G2N and G4N cells are named *B cells* (“*Proliferating*” cells) and G4P cells are named *C cells* (“*Citokine-producing*” cells)

While some groups of genes increase or decrease apparently continuously across the four RNA-seq data sets from G0N to G2N, G4N and G4P, there are also groups of genes that have non-monotonic patterns of expression (Fig. 2a). Therefore, it is unclear whether the differentiation is occurring through a single gradual progression or via discrete intermediate states.

We analyzed differentially expressed genes (DEGs) between subgroups and found roughly 1500 DEGs between G0N and G2N and between G4N and G4P but only 170 between G2N and G4N (Fig. 2b). Gene Ontology (GO) enrichment analysis (Fig. 2c) showed that G0N–G2N DEGs are enriched in “ATP biosynthetic process”, “Mitosis” and “Cell cycle”. The majority of these genes (70 to 85 %) are upregulated in G2N versus G0N, providing further confirmation that G0N cells are not actively proliferating. At the same time, the high expression of the activation marker *Cd69* [13] and the levels of *L-selectin* (*Cd62l*, *Sell*) and *Cd44* [14] in G0N cells and the increase in size of some of these cells (Additional file 4: Figure S2a, b, respectively) indicate that they have been partially activated so are no longer naïve cells.

Our GO analysis of DEGs between G4N and G4P indicated that the terms “Regulation of cytokine secretion”, “Cytokine activity” and “Regulation of T cell proliferation” represent the main categories of genes that are specifically differentially expressed together with *Il13* transcription (Fig. 2c). This means that the expression of *Il13* coincides with the expression of other genes important for Th2 function (*Il3*, *Il4*, *Il5*, *Il6*; Additional file 4: Figure S2c).

We also analyzed the expression changes of genes belonging to three important categories: signaling ligands (SLs), surface molecules (SMs), transcription factors (TFs) and cell cycle genes (Additional file 4: Figure S2d). Among the upregulated TFs we found some genes for which a role in Th2 differentiation has already been demonstrated (*Gata3*, *Batf3*, *Epas1* [15]) and some where their function still remains to be to elucidated (e.g. *Jdp2*, *Vdr*). In the SM group we observed the induction of *Il2ra* and *Il7r*, which are known to be involved in lymphocyte differentiation [16]. Moreover, the strong downregulation of *Ifngr1* observed in conjunction with cell activation is consistent with previous reports [17]. Overall, the vast majority (~75 %) of the differentially expressed SLs are upregulated from one generation to the next, including all the type 2 SLs.

Within the upregulated genes, we calculated the average Z-score across conditions for each of the four populations, as visualized in Fig. 2d. TFs and SMs are promptly upregulated soon after entering the cell cycle (G2N) and no further increase is detected after further cell division. SMs show a second prominent increase in their expression in cells when *Il13* is also expressed. No important effect of entering the cell cycle is visible on cytokine expression levels and only the production of *Il13* correlates with the expression of all SLs. Importantly, all the Th2-specific cytokines follow the same pattern of expression, continuing to be lowly expressed after entering the cell cycle and only undergoing a sharp boost from G4N to G4P.

Finally, the expression level of cell cycle genes is strongly upregulated from G0N to G2N, as expected by definition for these two subpopulations. More interestingly, we observed a second sharp increase in the expression of cell cycle genes in G4P cells, emphasizing the concomitant upregulation in cell cycle and differentiation genes, as we already observed from ex vivo Th2 single-cell RNA-seq. Finally, the expression of the Th2 signature increases from G0N to the consecutive negative generations and further increases from G4N to G4P (Fig. 2e). This group of genes includes most of the genes with a role in Th2 specification (*Ecm1*, *Il24*, *Batf*, *Il10*, *Nfil3*, *Gata3* and *Il4ra*) [18–20].

In summary, these data suggest that the G0N to G2N/G4N transition represents the exit from cell cycle arrest and entry into a proliferative cell state. The difference between G4N and G4P must result from a second major switch, which represents differentiation to the Th2 effector state with expression of the characteristic cytokines, including IL13*.* Together with expression of these cytokines, there is a parallel further increase in the expression of cell cycle genes.

The combination of the above results leads us to characterize Th2 cell differentiation as consisting of three major transcriptionally distinct states, which we name state A (activated cells that correspond to G0N), B (proliferating cells that correspond to both G2N and G4N) and C (cytokine expressing cells that correspond to G4P) (Fig. 2f).

### Validation of the three-state model at single-cell resolution

**Fig. 3:**
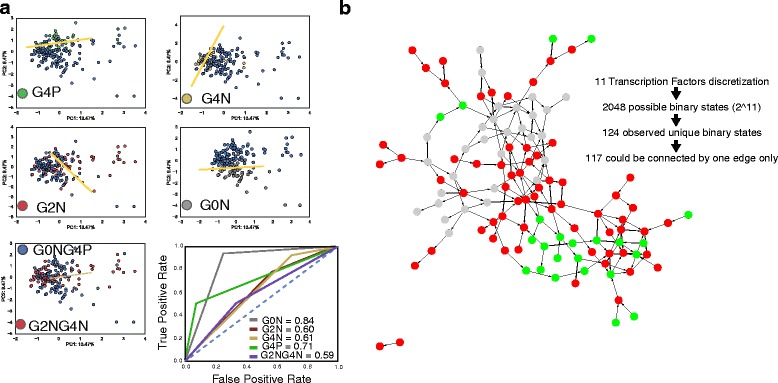
Single-cell qPCR analysis confirms three discrete states. **a** Linear principal component analysis (PCA) with a linear support vector classifier (SVC the yellow line) trained with “one-hot” labels (e.g. is it G4N or not) for each of the conditions and the first two principal component values were used to separate each of the generation and G2N/G4N cells from the other cells (in blue). The accuracy score of the ROC is reported for each individual generation and also for G2N/G4N (a traditional academic point system for accuracy scores can be found in Additional file 2). **b** State graph of 117 connected binary cell states for 11 transcription factors, constructed using the SCNS toolkit. Each *edge* represents the change in expression of a single gene. Grey circles are G0N cells, red circles are G2N/G4N cells and green circles represent G4P cells

Our description of three cell states during Th2 differentiation comes from population mRNA-seq data. Therefore, we aimed to verify our hypothesis at single-cell resolution by performing high-throughput single-cell qPCR analysis with dozens of genes in parallel in 46 cells from each population. We obtained a good overall correlation between the RNA-seq data and the average of the single-cell qPCR results (r2 = 0.78; Additional file 5: Figure S3a, b). Based on these data, we aimed to assign each cell to one of the three specific states we identified. We employed principal component analysis (PCA) and, to quantify the separation, a linear support vector classifier (SVC) was trained using “one-hot” labels (e.g. is it G4N or not) for each of the conditions and the first two principal component values (Fig. 3a). What we observed is that the SCV vector is able to distinguish G0N and G4P from the other cells with good and fair accuracies (scores are 0.83 and 0.72, respectively). Conversely, it fails to distinguish G2N and G4N states from the other cells and also the mixture of G2N and G4N from the rest of the cells (Fig. 3a; a traditional academic point system for accuracy scores can be found in Additional file 2). This analysis supports the existence of three states represented by G0N, G4P and a mixture of G2N and G4N.

In order to probe the transcriptional regulation of these states on a single-cell basis, we focused on 11 highly expressed transcription factors (Epas1, Myb, Mycn, Jhdm1d, Pou6f1, Pparg, Tcf7, Txk, Zc3h12c, Zfp36, Hlx) and discretized them into “on” or “off” states in each cell. This yielded 124 unique binary states and 117 of these can be connected by single-gene changes to yield a state graph as in Moignard et al. [21] (Fig. 3b). From this analysis we could observe that cells of the same state share similar TF organization as they cluster close to each other, underlining how TFs could act as master regulators of cell fate. We could also verify that the differentiation from A (in grey in Fig. 3b) to C (in green in Fig. 3b) requires the transition through at least one B cell (in red), further confirming the intermediate nature of this cell state. Taken together, these data confirm the concept of a three-state model of differentiation during Th2 primary activation. We also quantified the homogeneity of cells belonging to each of the three states as the average of Spearman cell-to-cell correlation (*p* value < 0.05) within each state (Additional file 5: Figure S3c). We observed an increase in the correlation across cells belonging to state C with respect to states A and B. This suggests that A and B cells are flexible and heterogeneous after primary activation, both before and after entering the cell cycle. In contrast, the cells that are more differentiated are more similar to each other, representing a more homogeneous population. These results, in agreement with data from Arsenio et al. on CD8+ T cells [22], support the concept of a commitment toward a more specific state in concert with the expression of the specific cytokines.

### Single cell fate: mathematical modelling of three cell states quantifies the link between acceleration of proliferation and differentiation

**Fig. 4:**
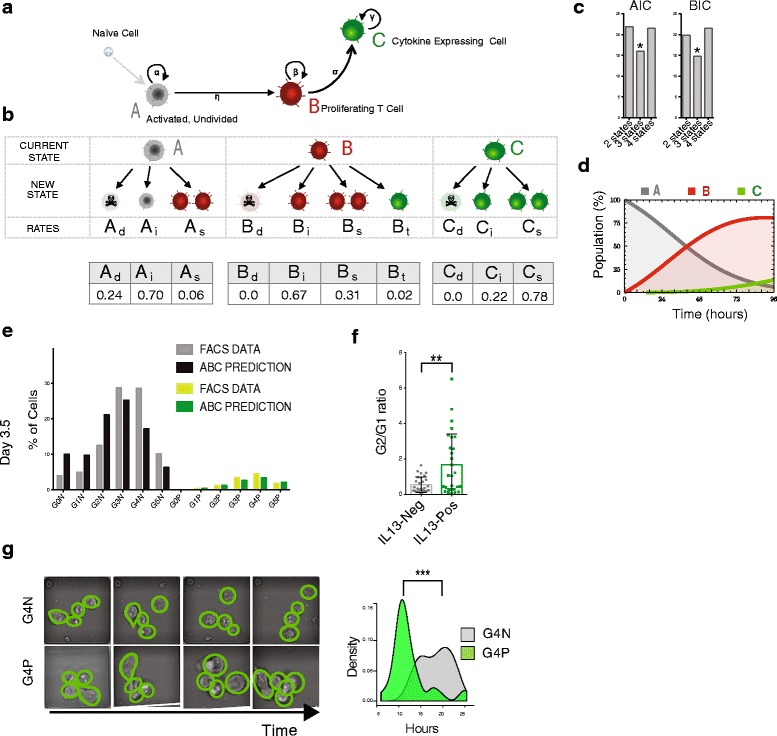
The model predicts T-cell behaviour at the cellular level. **a** Overview of the differentiation process that converts a naïve cell into a fully differentiated Th2 cell. Each naïve cell goes through three different states: state A (*Undivided*), state B (*Proliferating*) and state C (*Cytokine expressing*). **b** The model with examples of state-specific cell transitions and their corresponding probabilities. Transition probabilities are labelled as follows: *d* death, *i* stay identical, *s* symmetric division, *t* transdifferentiation. In the table, best fits of the model transition probabilities (expressed as probability per 14 h) from flow cytometry data at day 3.5 are reported. Data are representative of three independent mice **c** AIC and BIC for two-, three- and four-state models (the asterisks indicate the minimum values). **d** Cell subpopulations in the flow data at day 3.5 and the model prediction with parameters extracted at day 3.5. **e** Population dynamics of the three states over a 4-day period as predicted by the model. **f** The ratio between proportions of cells in G2 versus G1 is used as a measure of cell cycle speed when comparing both positive and negative cells within each individual generation (experiments are representative of four independent mice. Error bars indicate Standard Deviation, *p* value <0.01). **g** Live imaging of GEN4P versus GEN4N cells. Representative pictures from the live imaging time course experiment of G4N (*top*) and G4P (*bottom*) cells. Distribution of time of first division for G4N (*gray*) and G4P (*green*) cells (*p*-value <0.001)

To further dissect our three state hypothesis and verify and quantify the existence of a difference in the proliferation rate of different cells, we investigated the cellular (as opposed to molecular) events underlying cell differentiation across such states. We exploited flow cytometry data at day 3.5 of differentiation to discriminate between different models of cell differentiation. We considered a simple schematic, mathematical model of the behaviour of individual cells and their transformation dynamics across the three states A, B and C (Fig. 4a).

In our model, an activated naïve cell becomes an A cell that can then divide and give rise to B cells, which in turn can transdifferentiate into effector C cells. We assume that each cell can stochastically divide, die or differentiate into another state at given cell state-specific rates described by a Markov process (Fig. 4b). The death rate of an A cell in state A is named A_d_; and A_i_ is the rate at which the A cell remains identical. Since upon activation an A cell can start dividing, we consider the transition where an A cell divides symmetrically into two type B cells (rate A_s_). Analogously, a B cell can die (rate B_d_), stay the same (rate B_i_), duplicate (rate B_s_) or transdifferentiate into a type C cell (rate B_t_). T cells, similarly, can die, stay the same and divide (rates C_d_, C_i_ and C_s_).

The corresponding transition probabilities are shown in Fig. 4b (lower panel). The model, with the same parameters, also accurately models two additional fluorescence-activated cell sorting (FACS) datasets collected independently at days 2 and 3 of differentiation (Additional file 6: Figure S4a, b). We also calculated AIC (Akaike information criteria) and BIC (Bayesian information criteria) parameters for two-, three- and four-state models to verify whether our three-state model has the higher performance. AIC and BIC analysis supports the idea that in vitro Th2 primary activation is best described by three states (Fig. 4c).

Importantly, the day 3.5 fit appears to be robust because independent fits of day 2 and 3 data return fitting parameters very close to those of the day 3.5 fit (Additional file 2; Additional file 6: Figure S4c). Starting from the static picture of the system at day 3.5, our model predicts the detailed dynamics of the population composition (Fig. 4d). By solving the master equation of the model, the average number of A, B and C cells, as well as the composition of the subpopulation, can be derived at any time (Fig. 4e). By fitting FACS data at day 3.5, the values of the single cell transition probabilities can be determined (Additional file 2). The model fits our FACS data well in terms of the composition of the different cell subpopulations at day 3.5 (Fig. 4e).

With the parameters returned by the day 3.5 FACS data fit, the model predicts a twofold faster proliferation of C cells with respect to B cells, as expected from the gene expression profiles of cell cycle genes mentioned above. Interestingly, the differentiation rates of A and B cells (given by A_s_ and B_t_, respectively) are approximately one order of magnitude smaller than the growth rates of the populations of the three states, α, β and γ (Fig. 4b, e; Additional file 2; Additional file 6: Figure S4d).

It is worth noting that the predicted death rates of B and C cells are very small (B_d_ ≈ 0.0, C_d_ ≈ 0.0) during the first 3.5 days of differentiation. We were able to validate the difference of the death rate of A cells with respect to the other two states in independent experiments. Please note that apoptotic cells, measured as the sub-G1 DNA peak by Hoechst staining by flow cytometry, were only present in G0N cells, and completely absent in G1N, G2N, G3N and GFP-Positive cells (Additional file 6: Figure S4e).

To verify the higher division rate of differentiated cells, we also compared the cell cycle distribution of Hoechst-stained IL13-positive (C) and -negative cells (B) (Fig. 4f; Additional file 6: Figure S4f). Using the G2-M/G1 ratio as an indicator of the proportion of cycling cells, we observed that cells expressing *Il13* are cycling faster than the *Il13*-negative cells in the same generation (Fig. 4f), confirming our model predictions. Also, from the transcriptional point of view, cell cycle genes are highly upregulated in G4P compared with G4N cells (Additional file 6: Figure S4g), further suggesting an increase in cell cycle speed co-occurring with cytokine expression.

To give a more quantitative estimation of the cell cycle length in G4P versus G4N cells, we employed an automated imaging system to image single lymphocytes over a 20–40-h period. A MATLAB program, developed in house, was employed to extract the data from frames and the division time between mother and daughter cells was measured and tabulated (Fig. 4g). From the data, the average division time for G4P and G4N were computed to be 12.5 ± 4.2 and 18.7 ± 3.5 h, respectively (Fig. 4g).

These further experiments not only confirm the acceleration of cell division that occurs concomitantly with Th2 differentiation but also precisely quantify the difference in cell cycle length of T-h cells during primary activation.

### Asymmetric divisions and robustness of the model

We also tested an extended model including asymmetric divisions (named the “As” model; Additional file 2) in which we added the possibility of A and B cells dividing asymmetrically and giving rise to a B and a C cell (A_a_ and B_a_) (Additional file 6: Figure S4h, i). The As model does fit day 3.5 FACS data but it returns very low asymmetric transition rates in most of the different fits. This suggests that asymmetric transitions are extremely rare and, in fact, can be considered negligible with respect to symmetric ones (Additional file 2). These results are supported by the fact that when we considered a model (OA model) in which C cells can derive only by asymmetric division of A or B cells (A_s_ = B_t_ = 0), we obtain asymmetric transition parameters close to 0 (B_a_ ~ 0) and that C cells remain a very small fraction of the population even at long times, as γ < β (Additional file 6: Figure S4j).

We evaluated the AIC and BIC and found that both the AIC and BIC minima correspond best to the original model (Additional file 6: Figure S4k), i.e. the model without asymmetric division. Taken together, these results suggest that asymmetric transitions do not substantially contribute to Th2 differentiation.

### Validating the A, B and C cell states and parameters by expression profiling

Finally, we validated our model predictions with a dual experimental and computational approach. By combining the population dynamics predictions from the model with an RNA-seq data time-course, we aimed to link the cellular identity and the molecular characteristics of these cells.

First, we assigned a defined expression profile to each of the states: the G0N expression profile to A cells, the G2N profile to B cells and the G4P profile to C cells (Additional file 7: Figure S5a). Next, our model allows us to estimate the proportion of cells in each of the three states at different time points during Th2 differentiation (Fig. 4f). Based on the expression profile of each of the states, we are able to predict ensemble transcriptomic profiles at different time points.

**Fig. 5:**
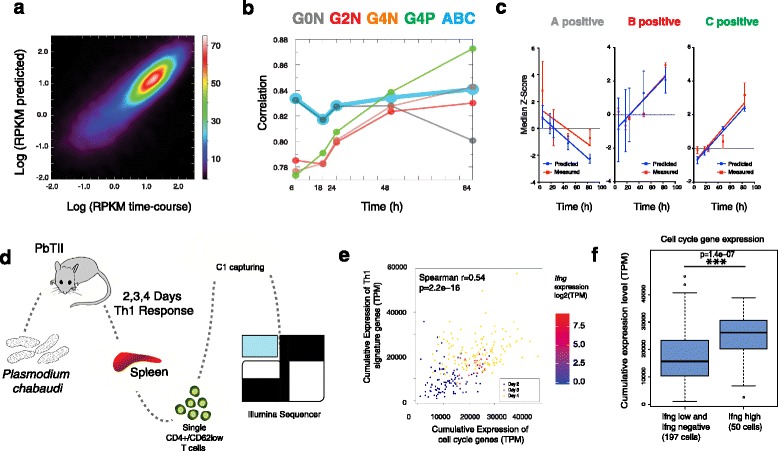
Model validation in vitro and in vivo. **a** Cell culture expression time course: correlations of gene expression levels (logRPKM) between the model prediction and measured data at 6, 18, 24, 48 and 84 h. The *colour scale* represents the density of transcripts as a percentage of all expressed genes. **b** Pearson correlation between model-predicted expression values (*thick light blue line*) and generation profiles (*thin lines*, *grey* for G0N, *red* for G2N, *orange* for G4N and *green* for G4P). **c** For each of the states, the linear regression of the median values (Z-score normalized) is visualized as a *red line* for the time-course data and as a *blue line* for the predicted ones. Errors were calculated using data from different RNA-seq replicates. **d** Overview of splenic Th cells isolated from PcAS PbTII-infected mice at 2, 3 and 4 days post-infection. **e** Cumulative expression levels of cell cycle-associated genes (Cyclebase) and Th1 signature genes [23] in single CD4+ T cells as TPM (transcripts per millions). Cells from different days are plotted with different *colours*. The *p* value was calculated using the cor.test function in R, based on the Spearman’s product moment correlation coefficient. **f** The cumulative expression of 251 cell cycle-associated genes as TPM (Cyclebase database) in Ifng high cells and in cells expressing low levels of or no Ifng. The *p* value was calculated using a Wilcoxon rank sum test

To verify the accuracy of our predictions, we performed a time-course mRNA-seq experiment (6, 12, 24, 48 and 84 h post-activation) in the same culture conditions used before (Additional file 7: Figure S5b). We analyzed the expression profiles of all the genes at all time points and plotted the predicted versus the measured log(RPKM) values (Fig. 5a). The correlation coefficient between the two was high (r ~ 0.83) and discrepancies were mainly at low expression levels, as expected.

Then we calculated the correlations between the single generation datasets, the model prediction and the new time-course data (Fig. 5b). As expected, the correlation of G0N decreases along consecutive time points while the correlation of G2N, G4N and G4P rises over time. Reassuringly, our predictions consistently have the highest correlation coefficients with the observed data over the whole time course (0.83 on average). Only the last time point (84 h) correlates better with G4P expression data. Globally, we observed that ~43 % of total genes have a correlation r > 0.5; if we consider only the DEGs, about ~60 % of them have r > 0.5 (Additional file 7: Figure S5c).

To further verify our prediction, we classified genes as negative or positive signatures, i.e. genes that are overexpressed in one state only (positive) and genes that are downregulated in one state only (Additional file 7: Figure S5d; see Additional file 7: Figure S5e for numbers of genes in each category and the “Methods” section for further details). To minimize the noise, we considered only the top 30 % of the significant positive signature genes, as those genes should be most representative of each particular cell state. We compared our prediction (Additional file 7: Figure S5f, bottom row) with the measured data (Additional file 7: Figure S5f, upper row). Inspection of the data indicated that the trend was consistent for each of the three states, with A-positive signature gene expression decreasing over time, while B and C gene levels increase.

Linear regression on the median expression (Fig. 5c) for the predicted and the experimentally determined data behave similarly, suggesting that our predictions fit well for all three states across all time points. Overall, the excellent agreement between the predicted and the measured RPKM values (Fig. 5a–c) shows that our model is accurate in terms of transcriptomic changes in Th2 differentiation.

These results confirm not only the three cell states during Th2 differentiation but also the robustness of the cellular parameters inferred by the model.

### Single-cell RNA-seq links CD4+ T-cell division rates to differentiation state in an in vivo Th1 infection model

To verify the link between cell cycle speed and differentiation rate in vivo and to ask if the model can be extended from Th2 to Th1 differentiation, we studied the CD4+ T-cell response against *Plasmodium chabaudi* AS (PcAS). Antigen-specific PbTII CD4+ T cells (CD45.1) were transferred into wild-type CD45.2 recipients and recovered from spleens at days 2, 3 and 4 post-infection (Fig. 5d).

As a measure of differentiation status inferred from the single-cell RNA-seq data, we developed a differentiation score based on the expression of “Th1 differentiation signature genes” [23]. We used aggregated G2/M gene expression levels across 26 genes as a “cell cycle score” reflecting division rate (analogous to [24]). Both of them are reported as TPM (transcript per million) and gene lists are provided in Additional file 3 and 8. We excluded that differences between cells in the different time points are confounded by batch effects with multiple analysis. First, the ERCC content across the two replicates of the same day shows no major differences (Additional file 7: Figure S5g). We can only observe a difference in the ERCC distribution across different days, but this variation can be due to the change in cell size from day 2 to days 3 and 4 rather than ERCC degradation (detailed explanation in Additional file 2). Moreover, the total number of reads is similar across all samples (Additional file 7: Figure S5h) and PCA of total genes and ERCC (Additional file 7: Figure S5i,j) clearly shows that replicates of the same day are similar to each other.

These single-cell transcriptomics data and bioinformatics analysis supports an increase in proliferation in more mature, differentiated cells in vivo, as highlighted by the correlation between Th1 differentiation and cell cycle score (Fig. 5e). Furthermore, similarly to our approach with Th2 cells, we focused only on the specific expression of *Infg* as the main readout of Th1 cell differentiation. We first categorized cells in two subgroups: *Ifng-*low and *Ifng-*high cells (Additional file 7: Figure S5k). When analysing the cumulative expression of cell cycle genes in these two subpopulations, we again observed that *Ifng*-high cells were expressing significantly higher amounts of cell cycle genes compared with *Ifng*-low cells. This analysis, together with our previous results, confirms that our observation of a strong link between differentiation and cell cycle speed is conserved in different subtypes of T-h cells.

## Discussion

Analysing single cell transcriptomes of CD4+ T cells in a mouse model of a type 2 immune response, we noticed an inter-dependency between cell cycle rate and cytokine expression.

Starting from this observation, we link a cell’s history and state, obtained by flow cytometry analysis, with transcriptome-wide expression profiles. Thus, we infer specific probabilities of cell death, division and differentiation and associate these outcomes with transcriptomic profiles.

What is unique about our strategy is that we sort cells from a single time point, thus reducing variability in confounding factors and allowing the identification of genes with essential function in lymphocyte differentiation (Additional file 9: Figure S6). This strategy can be applied to other cell types in which the cell cycle and differentiation are known to be inter-dependent (e.g. Th1, Th17 CD4+ cells, CD8+ T cells, intestinal epithelial cells, skin cells).

Using this approach, we first employ traditional bulk RNA-seq to profile the gene expression dynamics among different generations of differentiated and undifferentiated Th2 cells. A deep analysis revealed three discrete cell states during the differentiation process from naïve cells to effector Th2 cells. We named these three states A (activated, undivided) cells, which correspond to cells that have not entered the cell cycle (G0N), B (proliferating) cells, which are cycling but not differentiated (G2N and G4N), and C (Th2-like), which are cells that are cycling and expressing the differentiation marker (G4P). We validated the existence of these distinct states at the single-cell level by high-throughput single-cell qPCR, which supported a clear separation of G0N and G4P cells from the other cells and a single state across G2N and G4N cells.

These gene expression analyses characterize the three cell states at the molecular level. We extracted the cellular properties of death, division and differentiation rates in each of the three states using a Markov process to model flow cytometry data. This confirmed a surprising new finding: that cell division rate increases in mature differentiated cells compared with activated cells as suggested by cell cycle gene expression patterns.

**Fig. 6:**
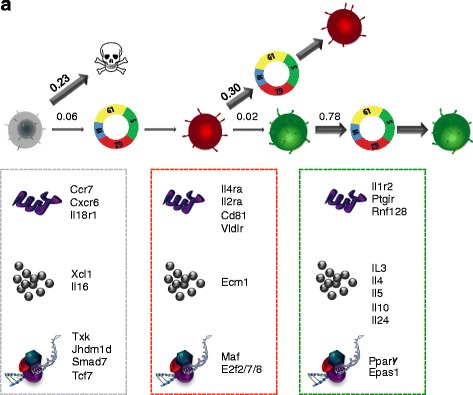
Schematic summary of the findings. Cells in state A can die or enter the cell cycle, transitioning into the B state. B cells can either cycle to give rise to more B cells or transdifferentiate into C state cells. C state cells can cycle giving rise to new C state cells. The thickness of the *arrows* corresponds to the likelihood of the event (rates are reported above the *arrows*). Each of the cell types is characterized by expression of specific sets of surface molecules (*top*), secreted factors (*middle*) and transcription factors (*bottom*) as summarized in the *box* below

Our findings at both the molecular and cellular levels are summarized in Fig. 6. Cells in state A have a high probability of dying but can eventually enter the cell cycle and give rise to cells in state B. During this process, the expression level of some TFs specifically expressed in A cells is downregulated and the expression of new TFs (i.e. *C-Maf* [25]) is switched on.

Amongst the A state-specific TFs are TFs with a known role in T-cell differentiation (e.g. *Txk* [26], *Smad7* [27]), along with several regulators not previously recognized in the context of Th cell differentiation (e.g. *Jhdm1d*, *Tcf7*). For example, *Xcl1* is known to act as a negative regulator of human CD4+ T-cell activation and to co-stimulate the apoptosis of human CD4+ T cells [28]. In our data, *Xcl1* is downregulated after entering the cell cycle, allowing type 2 CK production to take place and providing molecular insight into the higher death rate of A cells. Further cell cycle repressors highly expressed in the A state are *Ccr7*, known to inhibit T-cell proliferation [28], and *Il18r1*, known to inhibit Th2 development [29]. Concurrently, the A-to-B transition exhibits an increase in expression of *Il4ra*, *Il1r2*, *Ptgir*, *Rnf128* and *Itk*, in agreement with the literature [26, 30]. State C is characterized by the upregulation of several already known as well as previously uncharacterized transcription factors (i.e. *Epas1*, *Pparg* and *Jhdm1d*) in the context of Th2 differentiation. This new transcriptional network might contribute to the maintenance of a more defined and stable Th2 differentiated state, as observed by the higher cell-to-cell homogeneity of C cells compared with the other two states.

Globally, our data confirm many molecular studies in the literature and at the same time provide a more comprehensive and quantitative definition of gene regulation during Th2 differentiation from a new perspective. This high-resolution data set will provide a rich resource for further molecular mechanistic studies of T-h cell differentiation.

Finally, our mathematical model highlights important cellular characteristics of the three states. First, that undivided A cells have a higher death rate compared with activated cells. Second, that differentiated C cells have a higher division rate compared with B cells and that asymmetric divisions do not appear to contribute to the C state population. Live imaging of single lymphocytes not only confirmed our prediction but also gave a more robust quantification in terms of the difference in cell cycle length between cytokine-positive and -negative cells.

Moreover, despite being extracted from both in vivo and in vitro differentiated Th2 cells, our findings on the link between cell cycle speed and differentiation status are conserved in in vivo Th1 differentiating cells. In particular, we used single-cell RNA-seq data from a model of CD4+ T cells purified from PcAS PbTII-infected mice at 2, 3 and 4 days post-infection. Amongst the ex vivo cycling cells, we confirm a correlation between cell cycle speed and differentiation.

This finding supports a new concept during Th cell primary activation: not only is cell cycle entry a *sine qua non* for differentiation but a greater level of differentiation is associated with faster cycling cells—not only in Th2 cells but universally during primary activation of different subtypes of T lymphocytes.

Our findings suggest that cytokine-secreting mature T cells comprise the fastest cycling cell state as they are the most important cells for clearing an infection. Hence, the mechanism connecting cell division speed and cytokine secretion must have evolved for this purpose and may go awry in scenarios such as cytokine storms or, for instance, the severe immunopathology that can be associated with malaria. Insights into this process may be important to identify engineering strategies for T-cell therapy and targets for immunomodulation.

## Conclusions

In our work, we started from the observation that the type 2 cytokine-producing CD4+ T cells belong to the fast-cycling CD4+ T cells in a mouse model of *N. brasiliensis* infection. We inferred the accelerated proliferation from data mining of single cell RNA-seq transcriptomes. Next, we developed a new integrated multi-state modeling approach to dissect CD4+ T cell differentiation at both the cellular and molecular levels in an in vitro cell culture model. This showed that CD4+ T cell differentiation occurs through three major cell states, and that differentiated, cytokine-producing cells accelerate proliferation compared to early activated cells that are not expressing type 2 cytokines yet. We validated our findings at multiple levels: at single cell level using sc-RNA-seq, single cell qPCR and single cell live imaging, and at population level by bulk RNA-seq, as well as with conventional cell cycle analysis using DNA staining.

This finding supports a new concept of T helper cell primary activation: not only is cell cycle entry a *sine qua non* for differentiation, but a greater level of differentiation is associated with faster cycling cells. Based on similar observations in a type 1 response in a mouse model of malaria, this may well be a universal phenomenon during primary activation of CD4+ T lymphocytes.

The fast cycling cytokine-secreting mature T cells are likely key players for infection clearance. Hence the mechanism connecting cell division speed and cytokine secretion must have evolved for this purpose, and may go awry in scenarios such as cytokine storms or the severe immunopathology that can be associated with malaria. Insights into this process may have utility for engineering strategies for T cell therapy, and in identifying new targets for immunomodulation.

## Methods

### Ethics statement

Animal research at WTSI and LMB was conducted under license from the UK Home Office (PPLH 70/7968 and 70/8381, respectively) and used protocols approved by the institute’s animal welfare and ethical review body.

### *N. brasiliensis* infection

C57BL/6 female mice were subcutaneously injected with 100 μl (300/500 live third stage *N. brasiliensis* larvae per dose) over two sites. Mediastinal lymph nodes, mesenteric lymph nodes and lungs were taken from infected mice 5 days after infection. Cells were isolated from mediastinal lymph nodes and mesenteric lymph nodes by smashing the tissue though a 70-μm cell strainer. Lungs were incubated in collagenase D (0.72 mg/ml; Amersham, Bucks, UK) for 30 min, smashed though a 70-μm cell strainer and suspended in RBC lysis buffer (eBioscience Ltd). Single cells were then stained with CD3e-APC-cy7, CD4-PE-Cy7 and CD62L-Violet-BV510 for 30 min on ice and then washed.

### Single cell library preparation

For *N. brasiliensis*, three small (5–10 μm) C1 Single-Cell Auto Prep IFC chips (Fluidigm) were primed and 5000 cells were sorted directly into the chip. For *P. chabaudi*, cells were loaded at a concentration of 1700 cells μl^-1^ onto C1 small chips. To allow estimation of technical variability, 1 μl of a 1:4000 dilution of ERCC (External RNA Controls Consortium) spike-in mix (Ambion, Life Technologies) was added to the lysis reagent. Cell capture sites were visually inspected one by one using a microscope. The capture efficiency is described in Additional file 2: Tables S1 and S7.

The capture sites that did not contain single cells were noted and were removed from downstream analysis. Reverse transcription and cDNA preamplification were performed using the SMARTer Ultra Low RNA kit (Clontech) and the Advantage 2 PCR kit according to the manufacturer’s instructions on the C1 device. cDNA was harvested and diluted to 0.1–0.3 ng/μl and libraries were prepared in 96-well plates using a Nextera XT DNA Sample Preparation kit (Illumina) according to the protocol supplied by Fluidigm. Libraries were pooled and sequenced on an Illumina HiSeq2500 using paired-end 75-bp reads for *N. brasiliensis* and 100-bp reads for *P. chabaudi*.

### *P. chabaudi* infection

PbTII mice were housed under barrier conditions at the Wellcome Trust Sanger Institute. *P. chabaudi* wild-type strain 2.34 was maintained in female Theiler’s Original outbred mice and used in all experiments after one passage through naïve B6 mice. The 8–12-week-old female B6 mice were infected intravenously with 10^5^ PcAS-infected red blood cells and spleens were collected after cervical dislocation 2, 3 and 4 days later. For the day 2 and day 4 time points, samples from two mice were loaded onto two individual C1 chips for each day, while samples from one mouse were used for the intermediate time point.

### Cell culture

CD4+ cells were purified from *Il13-eGFP* homozygous BALB/C spleens from 6–12-week-old mice. After lympholyte-based (Cedarlane Laboratories) purification of lymphoid cells, magnetic negative selection (MACS, Miltenyi Biotec) allowed the purification of untouched CD4+ cells. Antibodies against CD8a, CD11b, CD11c, CD19, CD25 and Ly6G were used for depletion (see Additional file 2: Table S4 for details). The purity comprised between 90 and 96 %. Cells were cultured in Iscove's Modified Dulbecco's Medium (IMDM), 10 % fetal calf serum, 2 μM L-glutamine, penicillin, streptomycin and 50 μM β-mercaptoethanol. CellTrace Violet proliferation staining (Invitrogen) was performed according to the manufacturer’s instructions. Th2 polarization was induced with the standard protocol: cells were seeded at 1 × 10^5^/well in 96-well plates coated with anti-CD28 (4 μg/ml) and anti-CD3 (1 μg/ml) in the presence of IL4 (10 ng/ml; R&D Systems) and activated for 3.5 days in a humidified 37 °C, 5 % CO_2_ incubator.

### Bulk RNA-seq library preparation and qPCR

mRNA was isolated from two mice using an Oligotex Direct mRNA Mini Kit (Qiagen). Library preparation was performed according to the Illumina Single-End sequencing guide and sequenced using an Illumina Hi-Seq 2000 system. Total RNA was extracted with an RNeasy miniprep kit (Qiagen) according to the manufacturer’s instructions and reverse transcribed with SuperScriptIII (Invitrogen). cDNA was used for qPCR with specific primers and SYBR Green PCR Master Mix (Applied Biosystems) using an Illumina Eco Real-Time PCR System. A full list of primers can be found in Additional file 2: Table S5.

### Single-cell qPCR

Single-cell gene expression analysis was performed using the BioMark 96.96 Dynamic Array platform (Fluidigm, San Francisco, CA, USA) and TaqMan gene expression assays (Applied Biosystems, Carlsbad, CA, USA). We sorted 48 single cells for each of the four populations into 5 μl of CellsDirect reaction mix and these were immediately stored at −80 °C. Control wells containing no cells were included. Upon thawing, a mix containing 2.5 μL gene-specific 0.2× TaqMan gene expression assays (Applied Biosystems), 1.2 μL CellsDirect RT/Taq mix and 0.3 μL TE buffer were added to each well. RT-PCR pre-amplification cycling conditions were: 50 °C, 15 min; 95 °C, 2 min; 22 × (95 °C, 15 s; 60 °C, 4 min). Samples were diluted 1:5 in TE buffer and 6 % were mixed with TaqMan Universal PCR Master Mix (Applied Biosystems). The sample mix and TaqMan assays were loaded separately into the wells of 96.96 Gene Expression Dynamic Arrays (Fluidigm) in the presence of appropriate loading reagents. The arrays were read in a Biomark analysis system (Fluidigm). ΔCt values were calculated in reference to the average of Atp5a1, Hprt1 and Ubc. A list of the genes analysed can be found in Additional file 2: Table S6.

### FACS

Cells were sorted with a Beckman-Coulter MoFlo Cell Sorter and analysed on Fortessa or LSRII BD cell analyzers using FlowJo software. Surface and intracellular stainings were carried out according to the eBioscience protocols. A full list of the antibodies used is provided in Table S4 in Additional file 2.

### Hoechst staining

Cells were stained with Hoechst 33342 (Sigma Aldrich) at 5 μg/ml final concentration in complete medium for 60 min at 37 °C in a water bath. After washing they were re-suspended in cold PBS and run on the FACS analyser.

### Expression level calculation for bulk and single-cell RNA-seq data

Reads were mapped to the *Mus musculus* genome (Ensembl version 38.70) concatenated with the ERCC sequences using GSNAP with default parameters [31]. Gene-specific read counts were calculated using HTSeq (http://www-huber.embl.de/users/anders/HTSeq/). Read counts were normalized by gene length and RPKM (reads per kilobase of transcript per million reads mapped) values were calculated. The gene lengths were calculated as the union of all exons within a gene based on the Ensembl annotation. For bulk data differential expression analysis was performed in R using the DESeq package [32] with default parameters (Benjamini–Hochberg adjusted *p* value (false discovery rate) <0.1). For single-cell RNA-seq only TPM (transcripts per million) were used to quantify relative abundance of gene expression. To exclude poor quality cells, we looked at the percentage of reads mapped to 37 genes on the mitochondrial chromosome as in [33]. For *Nippostrongylus* infection, 210 cells (out of 288) with a total number of detected reads mapping to exons lower than 100,000 or with more than 20 % of measured exonic reads corresponding to genes coded by the mitochondrial genome were excluded from further analyses. For *P. chabaudi* data, cells with detected transcripts for fewer than 2000 genes or with more than 35 % of measured endogenous TPM corresponding to genes encoded by the mitochondrial genome were excluded from further analyses.

### Comparison and clustering of generation profiles

Generation-to-generation distances were calculated using the function ‘dist’ of the freely available statistical software package ‘R’ (http://www.r-project.org/).

### Functional enrichment of DEGs

Functional enrichment (GO annotation) of DEGs was performed using DAVID 6.7.

### Cell to cell correlation

For each population, we first calculated pairwise Spearman correlation values between any two different cells and then took the average of the correlation values with *p* value <0.05.

### Principal component analysis

PCA plots of qPCR data were generated on expression data scaled to the unit interval using the Python package scikit-learn (citation instructions can be found at http://scikit-learn.org/stable/about.html#citing-scikit-learn). The lines separating classes were inferred by training a linear support vector classifier for the cells in their given classes. After removal of three cells, the expression data were logarithmically scaled by log(d + 1). To quantify the separation, a linear SVC was trained using “one-hot” labels (e.g. is it G4N or not) for each of the conditions and the first two principal component values. We consider the model score for the training data to be a measure for how distinct these classes are from each other. Accuracy is measured by the area under the ROC curve (traditional academic point system for accuracy scores: 0.90–1 = excellent; 0.80–0.90 = good; 0.70–0.80 = fair; 0.60–0.70 = poor; 0.50–0.60 = fail [34]). The analysis was performed using the Python library scikit-learn, in particular the classes sklearn.decomposition.PCA and sklearn.svm.LinearSVC.

### Live imaging

We employ a fully automated imaging system built around a Nikon Eclipse Ti-E inverted microscope using a Nikon Plan Apo \lambda 40× 0.95 N.A. dry objective and Grasshopper3 GS3-U3-23S6M-C camera from Point Grey Research. For bright field imaging a halogen lamp light source is used. For epi-fluorescence imaging we use a metal-halide lamp source and two fluorescence filter sets, violet (Semrock Inc FF01-390/40 excitation filter, FF416-Di01 dichroic filter, FF01-460/60 emission filter) and GFP (Semrock Inc. FF01-472/30 excitation filter, FF495-Di03 dichroic filter, FF01-520/35 emission filter).

For the experiment, three micro-well (50 μm by 50 μm; Microsurfaces)-containing imaging petri dishes were loaded with GEN4P and GEN4N, respectively. The dishes were placed in a custom-built environmental chamber at 37 °C, 5 % CO_2_ and 100 % humidity. After registering 50–100 single micro-well positions per each type of cell, the cell positions were imaged in bright field, violet and GFP channels for 20–40 h so that it is possible to observe one complete cell cycle.

A MATLAB program developed in house was employed to extract the data from frames and the division time between mother and daughter cells was measured and tabulated (Fig. 1).

### Data availability

RNA-seq data for the Th2 differentiation time course and at single generation resolution and Nb-infected scRNA-seq will be available in the ArrayExpress database (http://www.ebi.ac.uk/arrayexpress/) under accession numbers E-MTAB-3543 and E-MTAB-4619, respectively. The *P. chabaudi* data presented here are part of a larger study (manuscript under preparation). At the time of writing, the raw data files may not yet be available at ArrayExpress under accession E-MTAB-4388 as they may still be under embargo. Should you require access to the raw data files, please contact the corresponding author directly.

## Additional files

Additional file 1: Figure S1. Single cell RNA-seq controls and validation of the RNA-seq results at the RNA and protein levels. **a** The number of reads mapped to exons versus the proportion of reads mapped to mitochondrial genes is used to identify good quality cells (lower right quadrant) in single cells RNA-seq data from Nb infected mice **b** ERCC and housekeeping gene expression levels across all the cells **c** 78 single cells were clustered according to the expression of 7 proliferation marker genes from previous works. Three cells expressing IL4 clustered within the group of cells expressing high levels of those genes *p*-value = 0.001 (Fisher's exact test.) **d** IL4 detection does not correlate with total number of reads. **e** Violet versus IL-13PE staining FACS plot as obtained using traditional antibody staining after 4.5 days of culture. **f** Correlation coefficient (r2) of all the possible pairwise comparison between the generation samples. **g** qPCR on a distinct biological replicate for the selected genes. GAPDH is used for normalization. For the RNA-seq average normalized count values are reported. **h** Comparison between intracellular staining and RNA-seq data. Blue bars represent percentage of positive cells, while red bars are average normalized count values. (PDF 591 kb) Additional file 2: Supplementary information and Tables S1–S8. (DOCX 175 kb) Additional file 3: A table of the cell cycle genes. (CSV 402 kb) Additional file 4: Figure S2. Additional gene expression analysis supports the existence of three discrete cell states during Th2 differentiation. **a** Expression levels of activation markers *Cd69*, *L-selectin* and *Cd44*. **b** Forward scatter (*FSC*) of cells in different generations. **c** Expression levels of different interleukins between G4N and G4P. Asterisks indicate a DEG. **d** Hierarchical clustering of genes divided by functional categories. Genes (*rows*) are sorted in ascending order of distance from G0N to G4P. (PDF 1590 kb) Additional file 5: Figure S3. Additional analysis confirmed the existence of three discrete states at the single cell level. **a** Violin plots represent the distribution of selected genes in single cells. The insets show bulk RNA-seq results. **b** Correlation between bulk data (*x-axis*) and single-cell qPCR (100-ct mean; *y-axis*). Each gene is represented by a line linking its values in G0N and G4P. **c** Average of Spearman correlation values between any two different cells with *p* value <0.05 is reported for the indicated population. (PDF 1136 kb) Additional file 6: Figure S4. Model validation. **a** Flow cytometry plot of CellTrace Violet versus *Il13-eGFP* at days 2 and 3 of Th2 differentiation. **b** Cell subpopulations in the flow data at days 3 and 2 and the model prediction with parameters extracted at day 3.5. **c**, **d** Comparison of the parameters extracted from fits at days 2, 3 and 3.5 and comparison of the parameters extracted from flow cytometry data at days 2, 3 and 3.5. **e** The SubG1 fraction; representative of three experiments. **f** Superposition of Hoechst staining profiles on IL13-negative (*top*, in *black*) versus IL13-positive (*bottom*, in *green*) cells. Quantification of the single-cell cycle phases is reported. Experiments are representative of three independent experiments at three different time points (****p* value <0.0005). **g** Row Z-score heatmap of top G2/M marker genes from Cyclebase.org in G4N versus G4P. **h** Asymmetric division considered in the As and OA models. **i**, **j** The As (**i**) and OA (**j**) model-predicted dynamics of the population fractions of the three states over a 4-day period. **k** AIC and BIC evaluated for day 3.5 across different models. (PDF 8084 kb) Additional file 7: Figure S5. Additional molecular validation of the model predictions in a Th2 and a Th1 system in an in vivo setting. **a** To each state we assigned a specific expression profile as visualized here. **b** Experimental setup of the time-course expression profiling. At the time points visualized total cells have been collected and total mRNA sequenced. **c** Pearson correlation distribution between measured and predicted values for all genes and DEGs only. **d**, **e** Identification of positive and negative signature genes as specifically ON or OFF genes in that particular state and their relative abundances. **f** Time-course data and the predicted expression levels of the 30 % top positive genes. The median is shown as a *black solid bar*. **g**, **h** Distribution of ERCC reads and total reads across samples. **i**, **j** PCA for all the genes and ERCC genes only is presented to exclude the presence of batch effects. Cells from different days are labelled as *differently coloured numbers* to symbolize cells from each day and batch. **k** The expression of *Ifng* in 247 single CD4+ T cells at days 2, 3 and 4 post-infection with *P. chabaudi* AS (PcAS) infection. Based on the distribution of expression levels, the single cells were categorized as *Ifng-low* or *Ifng-high*, as indicated by the vertical red line. (PDF 11869 kb) Additional file 8: A table of the Th1 differentiation genes. (CSV 445 kb) Additional file 9: Figure S6. GO analysis comparing the standard time-course approach and a generation-based approach. GO enrichment analysis of DEGs found only with a classic time-course-profiling experiment (in *yellow*, on the *left*) compared with genes found only with a generation-based approach (in *blue*, on the *right*). GO enrichment of the common genes is reported at the *bottom*. (PDF 1450 kb)
