# Erratum to: Single cell analysis of CD4+ T cell differentiation reveals three major cell states and progressive acceleration of proliferation

**DOI:** 10.1186/s13059-016-0998-9

**Published:** 2016-06-22

**Authors:** Valentina Proserpio, Andrea Piccolo, Liora Haim-Vilmovsky, Gozde Kar, Tapio Lönnberg, Valentine Svensson, Jhuma Pramanik, Kedar Nath Natarajan, Weichao Zhai, Xiuwei Zhang, Giacomo Donati, Melis Kayikci, Jurij Kotar, Andrew N. J. McKenzie, Ruddy Montandon, Kylie R. James, Daniel Fernandez-Ruiz, William R. Heath, Ashraful Haque, Oliver Billker, Steven Woodhouse, Pietro Cicuta, Mario Nicodemi, Sarah A. Teichmann

**Affiliations:** EMBL, European Bioinformatics Institute (EBI), Hinxton, CB10 1SD UK; Department of Physics, CNR-Spin, Istituto Nazionale di Fisica Nucleare (INFN), University of Naples Federico II, Napoli, Italy; Wellcome Trust Sanger Institute, Wellcome Trust Genome Campus, Hinxton, Cambridge CB10 1SA UK; Cavendish Laboratory, University of Cambridge, Madingley Road, Cambridge, CB3 0HE United Kingdom; Centre for Stem Cells and Regenerative Medicine, Kings College London, London, SE1 9RT UK; MRC Laboratory of Molecular Biology, Cambridge, CB2 0QH UK; QIMR Berghofer Medical Research Institute, Herston, Brisbane, Queensland Australia; Department of Microbiology and Immunology, The Peter Doherty Institute for Infection and Immunity, University of Melbourne, Melbourne, Victoria Australia; The Australian Research Council Centre of Excellence in Advanced Molecular Imaging, The University of Melbourne, Parkville, Australia; Department of Haematology, Cambridge Institute for Medical Research, University of Cambridge, Cambridge Biomedical Campus, Wellcome Trust/MRC Building, Hills Road, Cambridge, CB2 0XY UK; Wellcome Trust - Medical Research Council Cambridge Stem Cell Institute, University of Cambridge, Cambridge, UK

## Erratum

After the publication of this work [1] it was noticed that four authors were omitted from the author list and author contributions.

These authors have now been added to the list, and the updated author contributions are included below.

## Authors’ contributions

VP, LHV, KNN, JP and TL carried out ex vivo/in vitro experiments. RM and KRJ carried out in vivo malaria experiments under the supervision of OB and AH. VP, AP, VS, GK, GD, XZ, SW and MK performed data analysis. AP and MN carried out the mathematical modelling. DFR and WRH developed mouse model used in the study. AM provided mice and expertise on their use. WZ and JK carried out T-cell live imaging under the supervision of PC. VP, MN and SAT wrote the manuscript with contributions from all authors. SAT and MN supervised the work. All authors read and approved the final manuscript.

## Competing interests

The authors declare that they have no competing interests.
