# Supplementary figures and images for: Single-cell analysis of CD4+ T-cell differentiation reveals three major cell states and progressive acceleration of proliferation
Source: Genome Biol. 2016 May 12;17:103. doi: 10.1186/s13059-016-0957-5 (PMC4866375; doi:10.1186/s13059-016-0957-5)

Figure S1

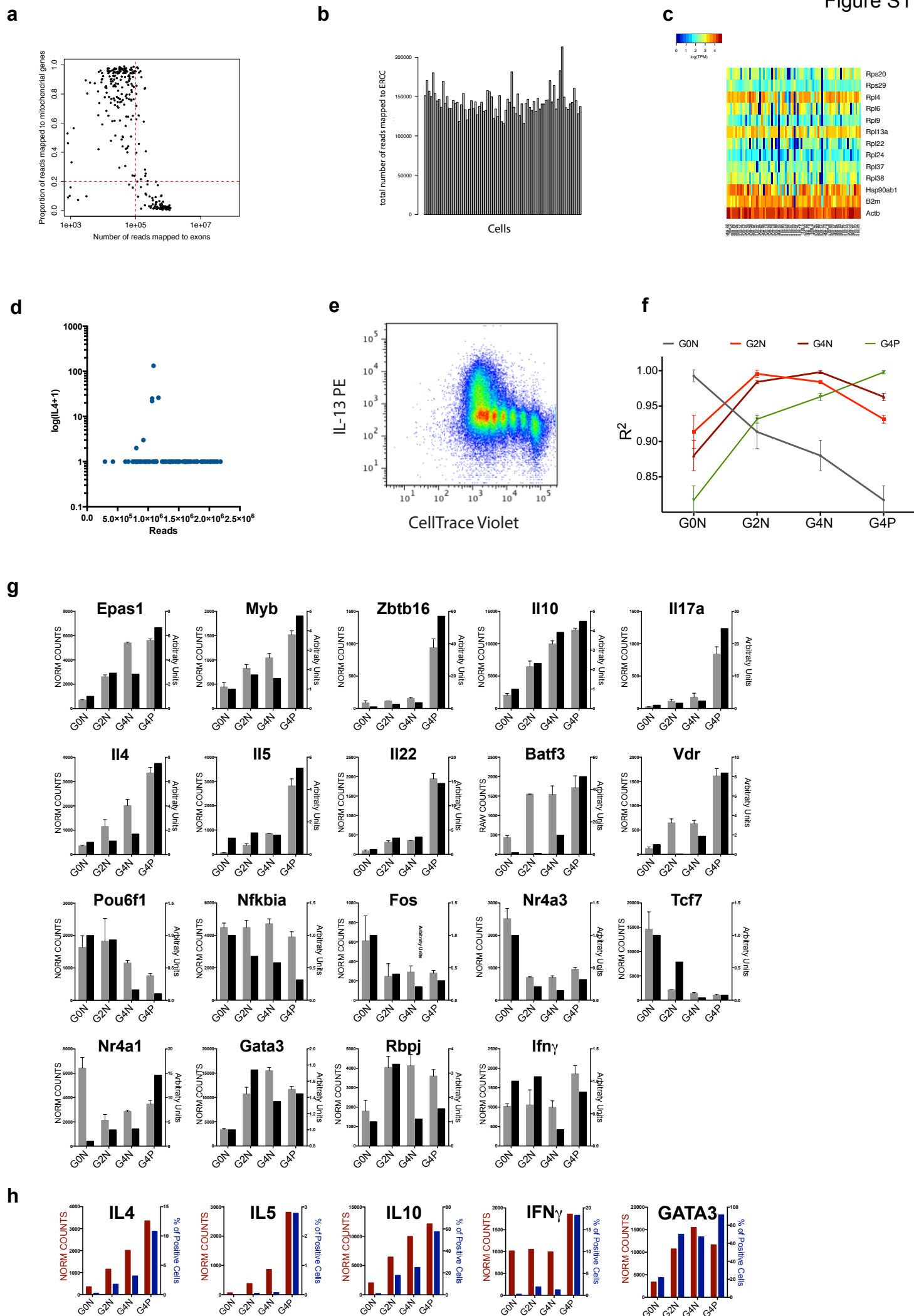

Supplement: Additional file 1: Figure S1. — Single cell RNA-seq controls and validation of the RNA-seq results at the RNA and protein levels. a The number of reads mapped to exons versus the proportion of reads mapped to mitochondrial genes is used to identify good quality cells (lower right quadrant) in single cells RNA-seq data from Nb infected mice b ERCC and housekeeping gene expression levels across all the cells c 78 single cells were clustered according to the expression of 7 proliferation marker genes from previous works. Three cells expressing IL4 clustered within the group of cells expressing high levels of those genes p-value = 0.001 (Fisher's exact test.) d IL4 detection does not correlate with total number of reads. e Violet versus IL-13PE staining FACS plot as obtained using traditional antibody staining after 4.5 days of culture. f Correlation coefficient (r2) of all the possible pairwise comparison between the generation samples. g qPCR on a distinct biological replicate for the selected genes. GAPDH is used for normalization. For the RNA-seq average normalized count values are reported. h Comparison between intracellular staining and RNA-seq data. Blue bars represent percentage of positive cells, while red bars are average normalized count values. (PDF 591 kb) [file 13059_2016_957_MOESM1_ESM.pdf]

a

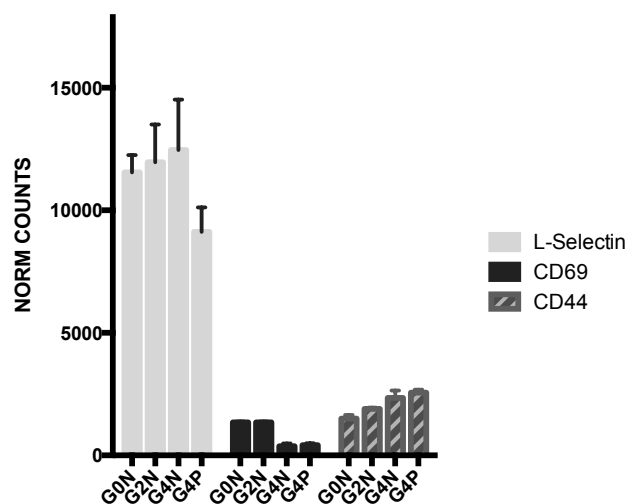

b

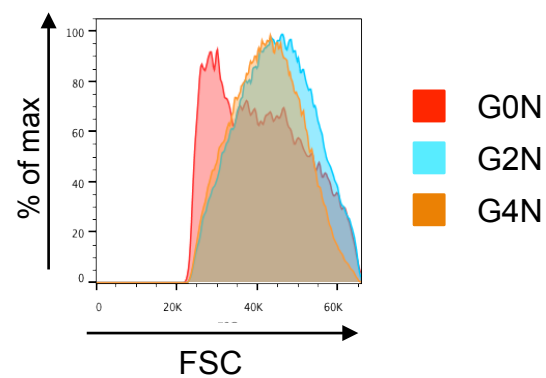

c

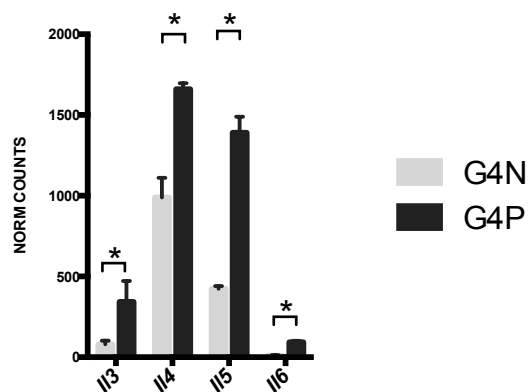

d

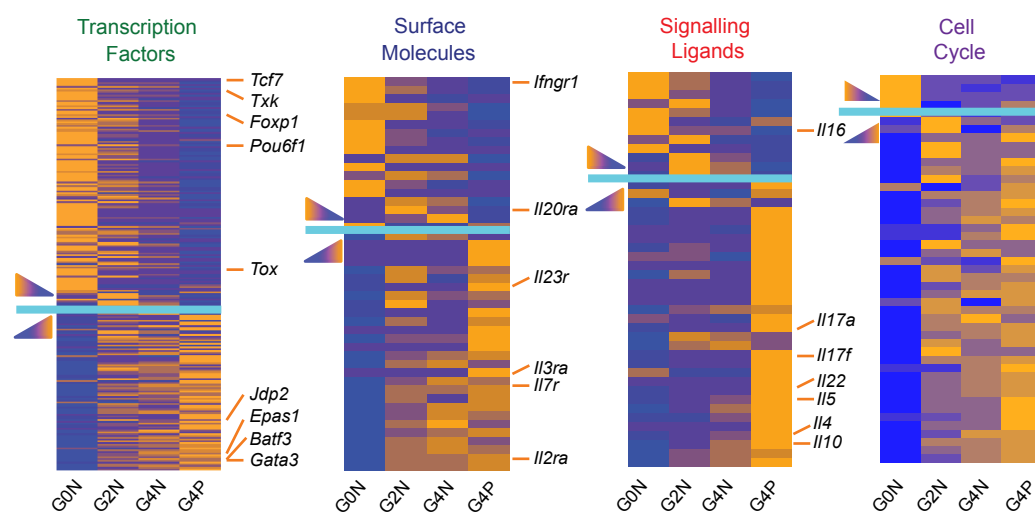

Supplement: Additional file 4: Figure S2. — Additional gene expression analysis supports the existence of three discrete cell states during Th2 differentiation. a Expression levels of activation markers Cd69, L-selectin and Cd44. b Forward scatter (FSC) of cells in different generations. c Expression levels of different interleukins between G4N and G4P. Asterisks indicate a DEG. d Hierarchical clustering of genes divided by functional categories. Genes (rows) are sorted in ascending order of distance from G0N to G4P. (PDF 1590 kb) [file 13059_2016_957_MOESM4_ESM.pdf]

**a**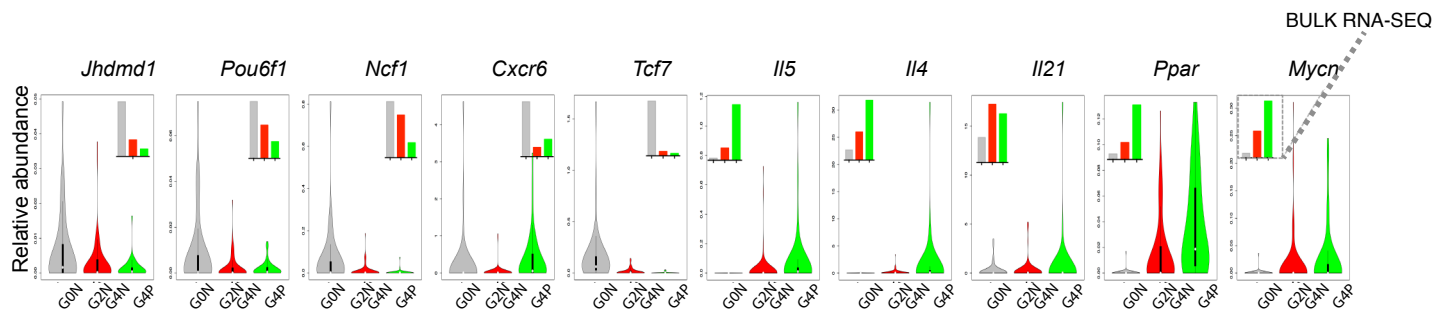**b**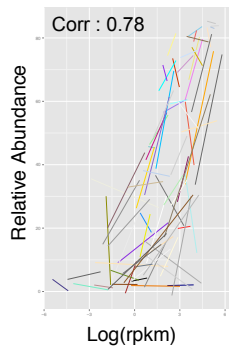**c**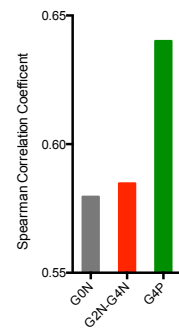

Supplement: Additional file 5: Figure S3. — Additional analysis confirmed the existence of three discrete states at the single cell level. a Violin plots represent the distribution of selected genes in single cells. The insets show bulk RNA-seq results. b Correlation between bulk data (x-axis) and single-cell qPCR (100-ct mean; y-axis). Each gene is represented by a line linking its values in G0N and G4P. c Average of Spearman correlation values between any two different cells with p value <0.05 is reported for the indicated population. (PDF 1136 kb) [file 13059_2016_957_MOESM5_ESM.pdf]

Figure S4

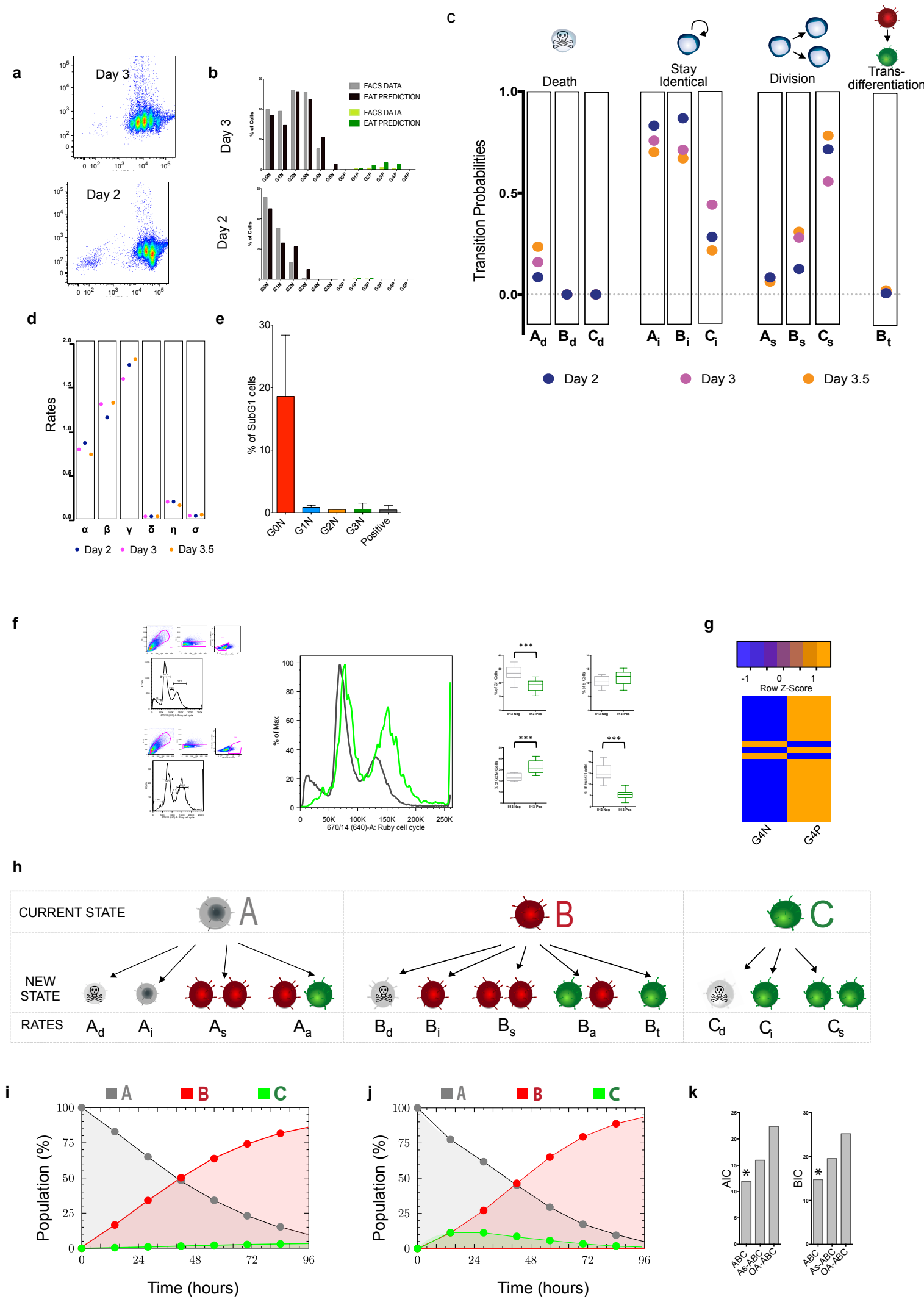

Supplement: Additional file 6: Figure S4. — Model validation. a Flow cytometry plot of CellTrace Violet versus Il13-eGFP at days 2 and 3 of Th2 differentiation. b Cell subpopulations in the flow data at days 3 and 2 and the model prediction with parameters extracted at day 3.5. c, d Comparison of the parameters extracted from fits at days 2, 3 and 3.5 and comparison of the parameters extracted from flow cytometry data at days 2, 3 and 3.5. e The SubG1 fraction; representative of three experiments. f Superposition of Hoechst staining profiles on IL13-negative (top, in black) versus IL13-positive (bottom, in green) cells. Quantification of the single-cell cycle phases is reported. Experiments are representative of three independent experiments at three different time points (***p value <0.0005). g Row Z-score heatmap of top G2/M marker genes from Cyclebase.org in G4N versus G4P. h Asymmetric division considered in the As and OA models. i, j The As (i) and OA (j) model-predicted dynamics of the population fractions of the three states over a 4-day period. k AIC and BIC evaluated for day 3.5 across different models. (PDF 8084 kb) [file 13059_2016_957_MOESM6_ESM.pdf]

Figure S5

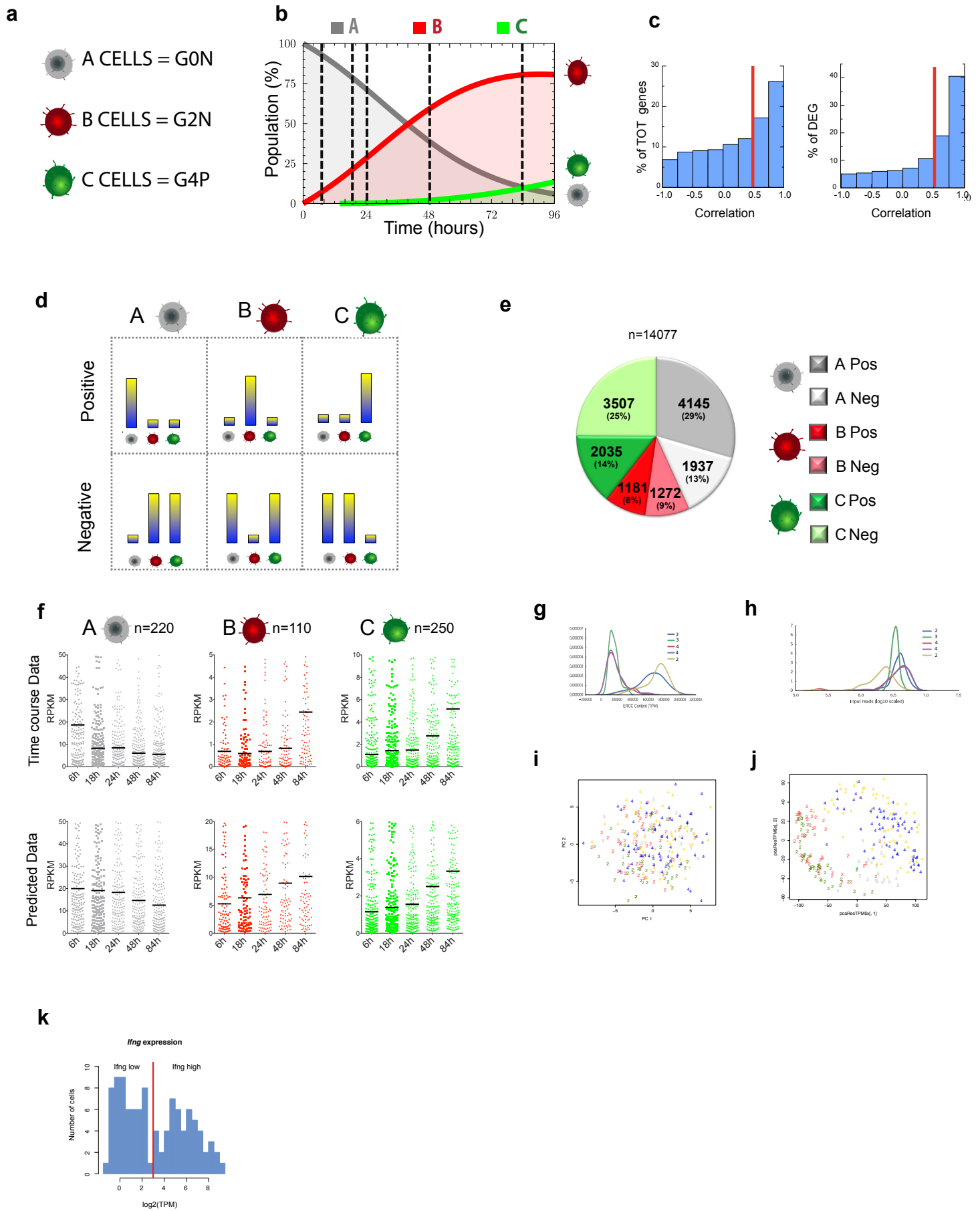

Supplement: Additional file 7: Figure S5. — Additional molecular validation of the model predictions in a Th2 and a Th1 system in an in vivo setting. a To each state we assigned a specific expression profile as visualized here. b Experimental setup of the time-course expression profiling. At the time points visualized total cells have been collected and total mRNA sequenced. c Pearson correlation distribution between measured and predicted values for all genes and DEGs only. d, e Identification of positive and negative signature genes as specifically ON or OFF genes in that particular state and their relative abundances. f Time-course data and the predicted expression levels of the 30 % top positive genes. The median is shown as a black solid bar. g, h Distribution of ERCC reads and total reads across samples. i, j PCA for all the genes and ERCC genes only is presented to exclude the presence of batch effects. Cells from different days are labelled as differently coloured numbers to symbolize cells from each day and batch. k The expression of Ifng in 247 single CD4+ T cells at days 2, 3 and 4 post-infection with P. chabaudi AS (PcAS) infection. Based on the distribution of expression levels, the single cells were categorized as Ifng-low or Ifng-high, as indicated by the vertical red line. (PDF 11869 kb) [file 13059_2016_957_MOESM7_ESM.pdf]

Supplementary Figure 6

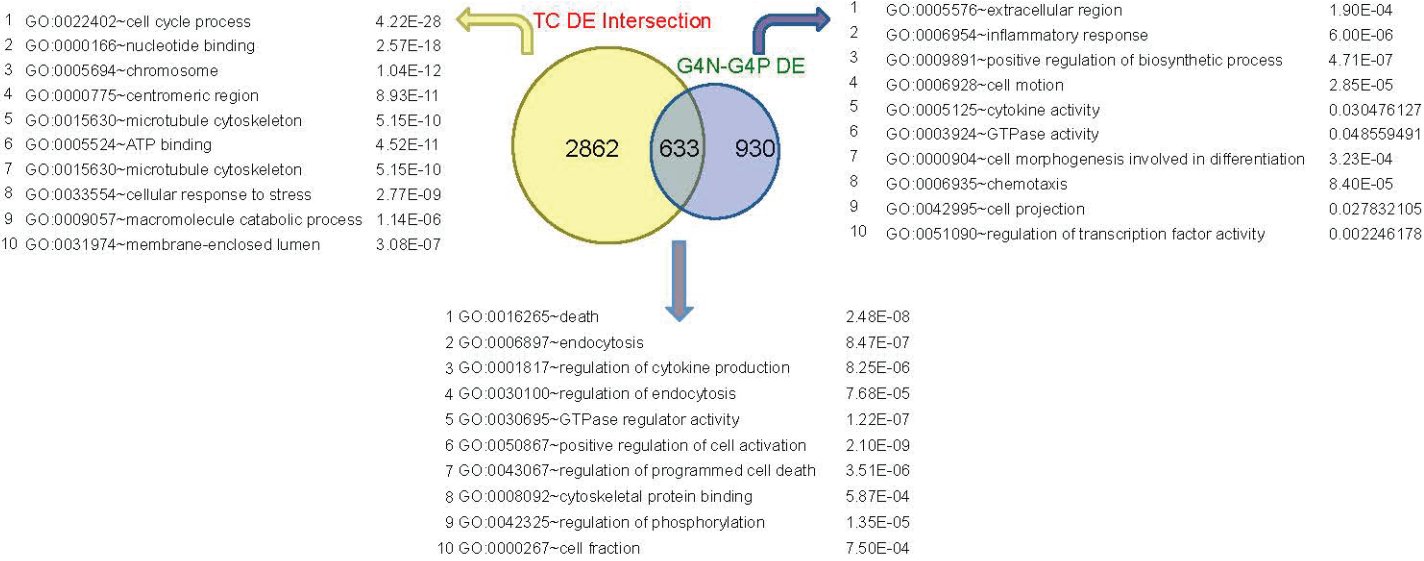

Supplement: Additional file 9: Figure S6. — GO analysis comparing the standard time-course approach and a generation-based approach. GO enrichment analysis of DEGs found only with a classic time-course-profiling experiment (in yellow, on the left) compared with genes found only with a generation-based approach (in blue, on the right). GO enrichment of the common genes is reported at the bottom. (PDF 1450 kb) [file 13059_2016_957_MOESM9_ESM.pdf]
