# Supplementary material for: Single-cell analysis of CD4+ T-cell differentiation reveals three major cell states and progressive acceleration of proliferation
Source: Genome Biol. 2016 May 12;17:103. doi: 10.1186/s13059-016-0957-5 (PMC4866375; doi:10.1186/s13059-016-0957-5)
Supplement: Additional file 2: — Supplementary information and Tables S1–S8. (DOCX 175 kb) [file 13059_2016_957_MOESM2_ESM.docx]

**Supplemental Information**

**The ABC model**

We model the cell dynamics as a stochastic Markov process which includes three states, named state A, B and C. For sake of mathematical simplicity, we considered a discrete time branching process, whose time step unit is named ∆t. We suppose that, from a time step to the next, an A cell can die (rate A_d_), stay the same (rate A_i_), divide giving rise to two type B cells (rate A_s_), or produce an asymmetric division in an B and a C cell (rate A_a_). A B cell can die (rate B_d_), stay the same (rate B_i_), duplicate (rate B_s_), give rise to a type C cell (rate B_t_), or divide asymmetrically in an B and a C cell (rate B_a_). Similarly, C cells can die, stay the same, divide (rates C_d_, C_i_, C_s_). A number of other state changes could be considered. For simplicity we only include the above ones which are motivated on biological grounds. In our FACS data at day 3.5 the last populated subgroup is G6N, which points out that six division steps must have occurred since day 0. From that we derive ∆t = 14h, a value compatible with the known duration of the cell cycle.

The ABC model can be analytically fully solved. The recursive relations of the average number of A, B and C cells, A(t), B(t) and C(t) can be derived from the Master Equation of the Markov process:

$$\left( \begin{matrix} A(t+1) \\ B(t+1) \\ C(t+1) \end{matrix} \right)=\left( \begin{matrix} \begin{matrix} \alpha\\ \eta\\ \delta\end{matrix} & \begin{matrix} 0 \\ \beta\\ \sigma\end{matrix} & \begin{matrix} 0 \\ 0 \\ \gamma\end{matrix} \end{matrix} \right)\left( \begin{matrix} A(t) \\ B(t) \\ C(t) \end{matrix} \right)$$

where

$\alpha=A_{i}$ ; $\beta=2B_{s}+B_{i}+B_{a}$ ; $\gamma=2C_{s}+C_{i}$ ; $\eta=2A_{s}+A_{a}$ ; $\sigma=B_{t}+B_{a}$; $\delta=A_{a}$.

The eigenvalues α, β, γ of the above matrix give the longer time scale growth rates of the A, B and C populations respectively.

The number composition of the different subpopulations, G0N, G1N, …, G6N can be analogously derived at any time, t, as a function of the microscopic parameters of the model.

**Fit of FACS data**

The initial condition considered in our fit corresponds to have all cells in state A, i.e., B(0) = T(0) = 0. The number of cells in the A population at day 0, A(0), is taken from the experiments. We refer here, for definiteness, to the case of the FACS data collected at day 3.5, where A(0) = 3.1 × 10^4^.

The model parameters are subject to the three normalization conditions of the probability rates in each single state. In the general case where asymmetric transitions are included, the ABC model has, thus, nine independent parameters. They are obtained by a Least Squares fit from the 13 measured subpopulations at day 3.5. More specifically, we find the minimum of the standard weighted squared distance:

$$D=\sum_{i=0}^{6} \omega_{\mathrm{iN}}\left( \frac{G_{\mathrm{iN}}}{G_{\mathrm{iN}}^{*}}-1 \right)^{2}+\sum_{i=0}^{5} \omega_{\mathrm{iP}}\left( \frac{G_{\mathrm{iP}}}{G_{\mathrm{iP}}^{*}}-1 \right)^{2}$$

where $G_{iN/P}$ and $G_{iN/P}^{*}$ are respectively, the predicted and observed number of IL13-GFP± cells at day 3.5 having undergone i divisions. Finally, in the expression for D the weights $\omega_{iN/P}$ are the relative fractions of the different observed populations: $\omega_{iN/P}=G_{iN/P}^{*}/\sum_{j} G_{jN/P}^{*}$.

**Positive and Negative State specific gene**

We call A Pos, B Pos and C Pos, those genes that were specifically expressed only in state A, B and C respectively. We named A Neg, B Neg and C Neg those genes whose expression was repressed only in state A, B and C respectively.

Genes were ranked with respect to the difference between the relative abundance in the positive state and the maximum relative abundance of the other states.

The relative abundance of a given gene i in state j, si,j is:

$$s_{i,j}=\frac{{RPKM}_{i,j}}{\sum_{j=E,A,T} {RPKM}_{i,j}}$$

where RPKMij is the RPKM value of gene i in state j.

In detail, we considered Positive only those genes whose expression is above their average expression only in one state (i.e, say s_i,j_>1 only in one state), while Negative genes are those whose expression is below their average expression only in one state (i.e, say s_i,j_<1 only in on state).

**Robustness of the fit with the ABC model**

In the main text we focused, in particular, on our FACS data at day 3.5 as they provide the broader and more precise measure of the different cell subpopulations. However, to asses the quality of our fit, we also considered independent FACS data at day 2 and day 3. Also in such cases the ABC model fits well the data. Importantly, the values of the parameters from the day 3.5 fit are, within errors, fully consistent with the values obtained by averaging the parameters of the fits across day 2, 3, 3.5. This is illustrated in the Figure S4c. Moreover, the fit of day 3.5 well reproduces day 2 and day 3 as shown in Figure S4b.

**Asymmetric divisions**

We also tested ABC model including asymmetric divisions. In particular, we considered a model (As-ABC) where asymmetric division (A_a_ and B_a_, see Figure S4h) are added and a model (OA-ABC) where transitions A_a_ and B_a_ are take into account instead of A_s_ and B_t_.

The OA-ABC and A-ABC models do fit the day 3.5 and day 3 and day 2 FACS data, but the state population growth curves obtained with the OA-ABC and As-ABC are markedly different from the one obtained with the ABC Model (i.e., model with A_a_ = B_a_ = 0), since the A population is predicted to dominate at all large time scales (see Figure S4i and j). In facts, in all cases the OA-ABC fits give, for the longer time scale, a growth rate for state B larger than for state C: β = 1.3 > γ = 0.2 (average). More importantly, OA-ABC finds that transition from B to C are absent (B_t_ = B_a_ = 0). The As-ABC gives in average β = 1.2 > γ = 0.9, but it results γ < β only in fit of day 2 and day 3 showing instability in model predictions. Moreover, the asymmetric models give negligible asymmetric transition parameters in some cases suggesting that asymmetric transitions are absent with respect to the symmetric one, in particular the As-ABC finds the same parameters of those found by the ABC model at day 3.5 with asymmetric transition completely absent (A_a_ = B_a_ = 0).

**Statistical Model Selection: AIC and BIC**

To compare the quality of the fit of FACS data at day 3.5 with different ABC models, we computed the corresponding Akaike Information Criterion (AIC) and Bayesian Information Criterion (BIC) model selection parameters. Let L_M_ be the log-likelihood of the parameter given the dataset, k the number of parameters and n the sample size. In this way AIC and BIC, for a model, are calculated through their definitions:

$$AIC=2k-2L_{M}$$

$$BIC=-2L_{M}+kln(n)$$

In particular, we compared the AIC and BIC of the ABC model with As-ABC and OA-ABC models. AIC and BIC were calculated for all three cases of day 2, day 3 and day 3.5. In all cases the Symmetric ABC Model represents the minimum, while the OA-ABC results to be always the maximum. This suggest that the ABC model shows the best performances in terms of model fitting and complexity of the model compared with As-ABC and OA-ABC as shown in Figure S4k, which is the case of the fit of day 3.5.

**ERCC total reads**

The reduction in ERCC content from day 2 to days 3 and 4 can be ascribed to changes in cell sizes and total mRNA content due to cell cycle activation (rather than ERCC degradation, which is an alternative explanation). Cells at day 2 have a diameter of roughly 6 μM, while at day 3 and 4 they can reach up to 10 μM of diameter. The corresponding cell volume can then vary by up to a factor of 5 with the result of a significant dilution of the ERCC molecules as in the plot below.

**Supplementary Table 1** Single cell Read Statistic and capturing efficiency for *Nippostrongilus brasiliensis* infection experiment

| **Cell Name** | **Total Reads** | **Mapped (%)** | **Unmapped (%)** | **Mapped to ERCCs (%)** |
| --- | --- | --- | --- | --- |
| **lung_3** | 1487939 | 80.5 | 19.0 | 20.0 |
| **lung_9** | 1928230 | 88.3 | 12.0 | 5.0 |
| **lung_19** | 2053091 | 79.3 | 21.0 | 8.0 |
| **lung_21** | 1610331 | 83.5 | 17.0 | 7.0 |
| **lung_27** | 930412 | 67.8 | 32.0 | 33.0 |
| **lung_43** | 1257027 | 73.4 | 27.0 | 24.0 |
| **lung_49** | 968646 | 68.1 | 32.0 | 28.0 |
| **lung_57** | 2184210 | 86.7 | 13.0 | 6.0 |
| **lung_58** | 2078589 | 82.4 | 18.0 | 6.0 |
| **lung_61** | 800147 | 73.5 | 27.0 | 31.0 |
| **lung_66** | 748231 | 76.4 | 24.0 | 39.0 |
| **lung_70** | 819601 | 87.7 | 12.0 | 8.0 |
| **lung_72** | 789447 | 87.2 | 13.0 | 3.0 |
| **lung_84** | 1748822 | 81.4 | 19.0 | 19.0 |
| **lung_94** | 1018096 | 84.1 | 16.0 | 11.0 |
| **lung_96** | 1446210 | 78.7 | 21.0 | 18.0 |
| **med_2** | 1504147 | 69.3 | 31.0 | 17.0 |
| **med_9** | 1645725 | 80.9 | 19.0 | 12.0 |
| **med_20** | 1063495 | 82.7 | 17.0 | 10.0 |
| **med_24** | 1358355 | 83.5 | 16.0 | 4.0 |
| **med_26** | 1372033 | 82.6 | 17.0 | 10.0 |
| **med_28** | 1491171 | 83.2 | 17.0 | 12.0 |
| **med_38** | 1805011 | 85.0 | 15.0 | 9.0 |
| **med_39** | 1283232 | 85.3 | 15.0 | 10.0 |
| **med_44** | 1110165 | 80.8 | 19.0 | 12.0 |
| **med_45** | 422249 | 80.1 | 20.0 | 16.0 |
| **med_46** | 674987 | 87.2 | 13.0 | 7.0 |
| **med_50** | 885442 | 81.0 | 19.0 | 16.0 |
| **med_53** | 1167662 | 85.2 | 15.0 | 9.0 |
| **med_54** | 961377 | 78.7 | 21.0 | 5.0 |
| **med_57** | 1656932 | 81.6 | 18.0 | 9.0 |
| **med_64** | 1361864 | 84.4 | 16.0 | 12.0 |
| **med_66** | 1133447 | 74.8 | 25.0 | 15.0 |
| **med_67** | 1065950 | 85.1 | 15.0 | 11.0 |
| **med_70** | 1515411 | 83.3 | 17.0 | 10.0 |
| **med_73** | 1603212 | 84.8 | 15.0 | 3.0 |
| **med_75** | 1570878 | 83.2 | 17.0 | 5.0 |
| **med_80** | 1613727 | 83.4 | 17.0 | 3.0 |
| **med_81** | 1605686 | 83.6 | 16.0 | 8.0 |
| **med_86** | 1087474 | 68.0 | 32.0 | 25.0 |
| **med_88** | 1674417 | 59.7 | 40.0 | 19.0 |
| **med_90** | 1377321 | 75.4 | 25.0 | 17.0 |
| **med_91** | 2129850 | 81.5 | 18.0 | 9.0 |
| **mes_4** | 1969331 | 86.9 | 13.0 | 8.0 |
| **mes_5** | 1894309 | 83.7 | 16.0 | 7.0 |
| **mes_11** | 2018354 | 84.2 | 16.0 | 9.0 |
| **mes_14** | 1590749 | 89.1 | 11.0 | 8.0 |
| **mes_15** | 1340324 | 83.5 | 17.0 | 13.0 |
| **mes_17** | 1228724 | 83.8 | 16.0 | 21.0 |
| **mes_19** | 2014018 | 88.8 | 11.0 | 7.0 |
| **mes_20** | 1491172 | 81.5 | 18.0 | 11.0 |
| **mes_21** | 1469916 | 76.6 | 23.0 | 9.0 |
| **mes_22** | 819325 | 77.5 | 23.0 | 25.0 |
| **mes_26** | 1097654 | 89.9 | 10.0 | 11.0 |
| **mes_29** | 1787702 | 88.1 | 12.0 | 10.0 |
| **mes_31** | 1867026 | 87.5 | 12.0 | 12.0 |
| **mes_34** | 1700704 | 88.5 | 11.0 | 10.0 |
| **mes_35** | 1241742 | 81.4 | 19.0 | 22.0 |
| **mes_36** | 2099841 | 82.3 | 18.0 | 8.0 |
| **mes_38** | 1001506 | 78.2 | 22.0 | 19.0 |
| **mes_44** | 922259 | 81.1 | 19.0 | 16.0 |
| **mes_51** | 1407907 | 89.2 | 11.0 | 11.0 |
| **mes_53** | 755217 | 68.5 | 32.0 | 38.0 |
| **mes_54** | 1075598 | 78.1 | 22.0 | 26.0 |
| **mes_56** | 1632849 | 82.5 | 18.0 | 12.0 |
| **mes_57** | 625455 | 86.4 | 14.0 | 7.0 |
| **mes_62** | 956076 | 85.3 | 15.0 | 11.0 |
| **mes_63** | 1135589 | 87.4 | 13.0 | 17.0 |
| **mes_64** | 1020401 | 81.1 | 19.0 | 20.0 |
| **mes_66** | 806268 | 82.1 | 18.0 | 30.0 |
| **mes_68** | 1790649 | 85.9 | 14.0 | 12.0 |
| **mes_72** | 1291221 | 79.1 | 21.0 | 18.0 |
| **mes_79** | 1338069 | 74.8 | 25.0 | 21.0 |
| **mes_80** | 858360 | 83.7 | 16.0 | 13.0 |
| **mes_84** | 1883627 | 72.9 | 27.0 | 13.0 |
| **mes_85** | 292537 | 82.7 | 17.0 | 13.0 |
| **mes_88** | 1254618 | 72.3 | 28.0 | 18.0 |
| **mes_93** | 1600476 | 83.1 | 10.0 | 10.0 |

Cell capturing efficiency

|  | Mediastinal  LN | | Mesenteric LN | Lungs | | Total (5) |
| --- | --- | --- | --- | --- | --- | --- |
| 1 cell | 60 | | 68 | 75 | | 71% |
| 0 cells | 0 | | 6 | 3 | | 3% |
| 2+ cells | 36 | | 22 | 18 | | 26% |
|  |  |  | | |  |  |

**Supplementary Table 2** Number of sorted cells and purity (between brackets)

|  | **G0N** | **G2N** | **G4N** | **G4P** |
| --- | --- | --- | --- | --- |
| **Replicate 1** | 74140 | 283389 (98%) | 665203 (97%) | 186135 |
| **Replicate 2** | 41079 | 236661 | 382689 | 93783 |

**Supplementary Table 3** Read statistic

Total number of reads, mapped reads (with relative percentage) and unmapped reads (with relative percentage). Spearman or correlations between biological replicates are reported.

|  | **Total** | **Mapp.** | **%** | **Unmapp.** | **%** | **Spearman Corr** | **Pearson Corr** |
| --- | --- | --- | --- | --- | --- | --- | --- |
| **G0Na** | 36389086 | 28801842 | 79.15 | 7587244 | 20.85 | 0.949 | 0.989 |
| **G0Nb** | 39659196 | 31818429 | 80.23 | 7840767 | 19.77 |  |  |
| **G2Na** | 55740953 | 47164694 | 84.61 | 8576259 | 15.39 | 0.959 | 0.991 |
| **G2Nb** | 50481720 | 40256442 | 79.74 | 10225278 | 20.26 |  |  |
| **G4Na** | 54573367 | 45166697 | 82.76 | 9406670 | 17.24 | 0.959 | 0.993 |
| **G4Nb** | 59346788 | 47739047 | 80.44 | 11607741 | 19.56 |  |  |
| **G4Pa** | 49367823 | 40873758 | 82.79 | 8494065 | 17.21 | 0.962 | 0.993 |
| **G4Pb** | 55327073 | 44434595 | 80.31 | 10892478 | 19.69 |  |  |

**Supplementary Table 4** Antibody list

| **Atibody** | **Conjugation** | **Clone** | **Company** |
| --- | --- | --- | --- |
| anti CD8 | FITC | Clone 536.7 | eBioscience |
| anti CD11b | FITC | Clone M1/70 | eBioscience |
| anti CD11c | FITC | Clone N418 | eBioscience |
| anti CD19 | FITC | Clone 1D3 | BD Bioscience |
| anti CD25 | FITC | Clone 7D4 | BD Bioscience |
| anti Ly6G | FITC | Clone RB6 8C5 | eBioscience |
| anti CD3e |  | Clone 1452C11 | eBioscience |
| anti CD28 |  | Clone 37.51 | eBioscience |
| anti GATA3 | Alexa647 | eBioscience | eBioscience |
| anti Tbx21 | PE | clone eBio4B10 | eBioscience |

**Supplementary Table 5** Primer list

| BATF3 | F | AGGACGATGACAGGAAAGTTCG |
| --- | --- | --- |
| BATF3 | R | CTGCAACCCGGTTTTTCTCT |
| EPAS11 | F | CTGAGGAAGGAGAAATCCCGT |
| EPAS11 | R | TGTGTCCGAAGGAAGCTGATG |
| FOS | F | CGGGTTTCAACGCCGACTA |
| FOS | R | TGGCACTAGAGACGGACAGAT |
| GAPDH | F | AACATCAAATGGGGTGAGGCC |
| GAPDH | R | GTTGTCATGGATGACCTTGGC |
| GATA3 | F | CCCTCCGGCTTCATCCTCT |
| GATA3 | R | CTGCACCTGATACTTGAGGC |
| IFNGAMMA | F | ATGAACGCTACACACTGCATC |
| IFNGAMMA | R | CCATCCTTTTGCCAGTTCCTC |
| IL-10 | F | TGGGTGAGAAGCTGAAGACC |
| IL-10 | R | GCTCCACTGCCTTGCTCTTA |
| IL-17a | F | CTCCAGAAGGCCCTCAGACTAC |
| IL-17a | R | AGCTTTCCCTCCGCATTGACACAG |
| IL-4 | F | ACTTGAGAGAGATCATCGGCA |
| IL-4 | R | AGCTCCATGAGAACACTAGAGTT |
| IL-5 | F | CTCTGTTGACAAGCAATGAGACG |
| IL-5 | R | TCTTCAGTATGTCTAGCCCCTG |
| IL-22 | F | AGACAGGTTCCAGCCCTACA |
| IL-22 | R | CTGGATGTTCTGGTCGTCAC |
| MYB | F | GAACAGATGGGCAGAGATCG |
| MYB | R | AGCCTTCCTGTTCCACCTTG |
| NFKBIA | F | ACGAGCAAATGGTGAAGGAG |
| NFKBIA | R | GGCTTCTCTTCGTGGATGAT |
| NR4A1 | F | GCAGTCTGTGGTGACAATGC |
| NR4A1 | R | CTCTTGTCCACAGGGCAATC |
| NR4A3 | F | ACTACGGAGTCCGCACCTG |
| NR4A3 | R | CTTGTCCACTGGGCAGTTTT |
| POU6F1 | F | GTCAGATCCTCACGAATGCTC |
| POU6F1 | R | GAGTCACGGCTTGGACCTG |
| RBPJ | F | CAGCAGCTGAACTTGGAAGG |
| RBPJ | R | GGAGGGTTTGGAGATGACCT |
| TCF7 | F | AGCTTTCTCCACTCTACGAACA |
| TCF7 | R | AATCCAGAGAGATCGGGGGTC |
| VDR | F | ACCCTGGTGACTTTGACCG |
| VDR | R | GGCAATCTCCATTGAAGGGG |
| ZBTB16 | F | GTGCCCAGTTCTCAAAGGAG |
| ZBTB16 | R | CCCACACAGCAGACAGAAGA |

**Supplementary Table 6** Biomark Gene list

Informative Genes

| Ahr |
| --- |
| Asb22 |
| Atf3 |
| Atp5a1 |
| Bcl2 |
| Bcl2l11 |
| Cd3e |
| Ptprc |
| Cdh17 |
| Cxcr3 |
| Cxcr5 |
| Cxcr6 |
| Cyp11a1 |
| Ddit3 |
| E2f8 |
| Epas11 |
| Foxp1 |
| Gata3 |
| Hlx |
| Ifng |
| Ifngr1 |
| Il10 |
| Il12rb2 |
| Il21 |
| Il4 |
| Il4ra |
| Il5 |
| Il6 |
| Il7r |
| Jhdm1d |
| Mllt6 |
| Myb |
| Mycn |
| Ncf1 |
| Pou6f1 |
| Pparg |
| Prdm1 |
| Ptgir |
| Pth |
| Rora |
| Rorc |
| Smad7 |
| Tbx21 |
| Tcf7 |
| Tg |
| Tgfb1 |
| Txk |
| Zc3h12c |
| Zfp36 |

Housekeeping genes

| Atp5a1 |
| --- |
| Ubc |
| polr2a |
| Elf2b |
| Hprt |

Lowly expressed/Not consistent with RNA-seq data genes

| Batf |
| --- |
| Fgf18 |
| Il2 |
| Il9 |
| Pde2a |
| Ccr8 |
| Sell |
| Runx3 |
| Il2ra |
| Msc |
| Ccr2 |
| Ccr5 |
| Cd4 |
| Eif2b1 |
| Gata2 |
| Il13 |
| Il13ra1 |
| Il23r |
| Il27r |
| Il4r |
| Irf8 |
| Phf1 |
| Scml4 |
| Vdr |
| Zbtb16 |

Negative Control Genes (other immune cell types/plant gene)

| Arf1 |
| --- |
| Hbb‑b2 |
| Cd33 |
| Cd19 |
| Cd20 |
| Cd8b1 |
| Cd56 |
| Cd11b |
| Cd11c |
| Cd44 |
| Foxp3 |

**Supplementary Table 7** Single cell Read Statistic for *Plasmodium chabaudi* infection experiment

|  | **Total Reads** | **Mapped (%)** | **Unmapped (%)** | **mapped to ERCCs (%)** |
| --- | --- | --- | --- | --- |
| Day2-1 | 4133223 | 96.7 | 3.3 | 71.9 |
| Day2-2 | 3435415 | 93.2 | 6.8 | 46.8 |
| Day2-3 | 3428253 | 95.0 | 5.0 | 48.9 |
| Day2-4 | 3269806 | 94.4 | 5.6 | 47.2 |
| Day2-5 | 2339041 | 88.6 | 11.4 | 11.6 |
| Day2-6 | 4778547 | 95.0 | 5.0 | 51.0 |
| Day2-7 | 3052057 | 92.0 | 8.0 | 13.2 |
| Day2-8 | 4206871 | 97.8 | 2.2 | 40.9 |
| Day2-9 | 3911806 | 95.9 | 4.1 | 49.2 |
| Day2-10 | 4052925 | 95.5 | 4.5 | 47.5 |
| Day2-11 | 4116936 | 91.3 | 8.7 | 33.0 |
| Day2-12 | 3001127 | 93.7 | 6.3 | 18.2 |
| Day2-13 | 4783089 | 91.2 | 8.8 | 37.8 |
| Day2-14 | 4058289 | 91.7 | 8.3 | 33.0 |
| Day2-15 | 3934260 | 93.1 | 6.9 | 22.4 |
| Day2-16 | 3971835 | 95.7 | 4.3 | 31.3 |
| Day2-17 | 3661468 | 95.8 | 4.2 | 62.5 |
| Day2-18 | 4595352 | 97.0 | 3.0 | 36.0 |
| Day2-19 | 2660478 | 93.5 | 6.5 | 34.7 |
| Day2-20 | 3632743 | 96.0 | 4.0 | 36.2 |
| Day2-21 | 3737703 | 94.3 | 5.7 | 37.6 |
| Day2-22 | 4391500 | 95.0 | 5.0 | 25.1 |
| Day2-23 | 3144777 | 98.0 | 2.0 | 55.6 |
| Day2-24 | 3524278 | 95.0 | 5.0 | 26.7 |
| Day2-25 | 2966082 | 94.4 | 5.6 | 34.4 |
| Day2-26 | 6310636 | 97.7 | 2.3 | 40.4 |
| Day2-27 | 4047191 | 96.0 | 4.0 | 53.2 |
| Day2-28 | 3889842 | 99.4 | 0.6 | 84.8 |
| Day2-29 | 4391290 | 94.1 | 5.9 | 38.7 |
| Day2-30 | 4757378 | 95.9 | 4.1 | 53.1 |
| Day2-31 | 4341656 | 95.3 | 4.7 | 26.4 |
| Day2-32 | 4055725 | 94.4 | 5.6 | 29.1 |
| Day2-33 | 5140121 | 94.7 | 5.3 | 35.0 |
| Day2-34 | 3389254 | 95.0 | 5.0 | 26.4 |
| Day2-35 | 3200548 | 97.3 | 2.7 | 76.5 |
| Day2-36 | 1874506 | 89.6 | 10.4 | 9.3 |
| Day2-37 | 1795653 | 89.6 | 10.4 | 18.4 |
| Day2-38 | 3039249 | 94.7 | 5.3 | 38.5 |
| Day2-39 | 3631534 | 97.1 | 2.9 | 39.7 |
| Day2-40 | 4008279 | 91.4 | 8.6 | 32.7 |
| Day2-41 | 5300814 | 92.0 | 8.0 | 41.3 |
| Day2-42 | 5055768 | 96.4 | 3.6 | 40.3 |
| Day2-43 | 5247577 | 96.0 | 4.0 | 41.2 |
| Day2-44 | 4195124 | 97.1 | 2.9 | 77.8 |
| Day2-45 | 3203341 | 94.8 | 5.2 | 23.1 |
| Day2-46 | 3856873 | 94.0 | 6.0 | 49.9 |
| Day2-47 | 3992316 | 96.5 | 3.5 | 57.7 |
| Day2-48 | 3293602 | 93.6 | 6.4 | 15.8 |
| Day2-49 | 4073607 | 93.6 | 6.4 | 33.9 |
| Day2-50 | 6795957 | 94.8 | 5.2 | 49.2 |
| Day2-51 | 5682346 | 95.7 | 4.3 | 54.3 |
| Day2-52 | 5657855 | 96.7 | 3.3 | 48.1 |
| Day2-53 | 4675838 | 94.7 | 5.3 | 44.8 |
| Day2-54 | 5226447 | 94.0 | 6.0 | 37.1 |
| Day2-55 | 3945055 | 94.0 | 6.0 | 27.1 |
| Day2-56 | 3277850 | 93.4 | 6.6 | 41.8 |
| Day2-57 | 4485815 | 97.9 | 2.1 | 34.1 |
| Day2-58 | 3534877 | 94.1 | 5.9 | 42.0 |
| Day2-59 | 4314973 | 89.6 | 10.4 | 33.6 |
| Day2-60 | 4135137 | 96.6 | 3.4 | 61.1 |
| Day2-61 | 3204662 | 90.4 | 9.6 | 22.5 |
| Day2-62 | 4604455 | 89.2 | 10.8 | 14.5 |
| Day2-63 | 2266048 | 97.2 | 2.8 | 74.1 |
| Day2-64 | 3146084 | 97.9 | 2.1 | 78.9 |
| Day2-65 | 3680954 | 94.8 | 5.2 | 34.9 |
| Day2-66 | 1814091 | 88.7 | 11.3 | 36.5 |
| Day2-67 | 904345 | 93.2 | 6.8 | 67.0 |
| Day2-68 | 2566002 | 90.0 | 10.0 | 47.5 |
| Day2-69 | 1871331 | 91.8 | 8.2 | 60.8 |
| Day2-70 | 1947833 | 92.3 | 7.7 | 58.7 |
| Day2-71 | 1535703 | 95.4 | 4.6 | 69.6 |
| Day2-72 | 1070463 | 96.5 | 3.5 | 70.4 |
| Day2-73 | 1569117 | 93.2 | 6.8 | 55.7 |
| Day2-74 | 1499146 | 90.2 | 9.8 | 43.2 |
| Day2-75 | 2290535 | 94.1 | 5.9 | 58.8 |
| Day2-76 | 913484 | 97.0 | 3.0 | 86.4 |
| Day2-77 | 2234854 | 90.1 | 9.9 | 41.4 |
| Day2-78 | 2675755 | 95.2 | 4.8 | 69.3 |
| Day2-79 | 761920 | 95.1 | 4.9 | 62.5 |
| Day2-80 | 2285398 | 89.8 | 10.2 | 45.6 |
| Day2-81 | 1771452 | 94.8 | 5.2 | 65.8 |
| Day2-82 | 1963853 | 93.0 | 7.0 | 56.9 |
| Day2-83 | 1969632 | 97.0 | 3.0 | 73.6 |
| Day2-84 | 873648 | 92.4 | 7.6 | 66.4 |
| Day2-85 | 1555037 | 93.9 | 6.1 | 63.9 |
| Day2-86 | 1361770 | 94.0 | 6.0 | 62.7 |
| Day2-87 | 1965443 | 87.3 | 12.7 | 39.0 |
| Day2-88 | 840884 | 93.1 | 6.9 | 63.5 |
| Day2-89 | 2078510 | 91.9 | 8.1 | 58.1 |
| Day2-90 | 2382656 | 92.4 | 7.6 | 51.7 |
| Day2-91 | 2136037 | 90.3 | 9.7 | 45.8 |
| Day2-92 | 1207217 | 92.7 | 7.3 | 61.6 |
| Day2-93 | 1587915 | 97.3 | 2.7 | 78.4 |
| Day2-94 | 1286158 | 94.4 | 5.6 | 72.9 |
| Day2-95 | 1810082 | 93.6 | 6.4 | 61.0 |
| Day2-96 | 168815 | 96.8 | 3.2 | 76.4 |
| Day2-97 | 2166049 | 94.7 | 5.3 | 65.9 |
| Day2-98 | 1437104 | 94.0 | 6.0 | 66.7 |
| Day2-99 | 2573322 | 91.9 | 8.1 | 50.2 |
| Day2-100 | 1607540 | 96.1 | 3.9 | 80.6 |
| Day2-101 | 1071741 | 94.4 | 5.6 | 72.1 |
| Day2-102 | 2368179 | 90.3 | 9.7 | 62.1 |
| Day2-103 | 3203672 | 86.6 | 13.4 | 27.3 |
| Day2-104 | 2317557 | 95.9 | 4.1 | 67.5 |
| Day2-105 | 3076419 | 87.4 | 12.6 | 35.3 |
| Day2-106 | 2601905 | 93.6 | 6.4 | 58.5 |
| Day2-107 | 3393576 | 85.4 | 14.6 | 22.1 |
| Day2-108 | 2148526 | 92.8 | 7.2 | 59.0 |
| Day2-109 | 2840076 | 95.3 | 4.7 | 66.2 |
| Day2-110 | 4569705 | 86.5 | 13.5 | 20.4 |
| Day2-111 | 2149176 | 95.7 | 4.3 | 68.8 |
| Day2-112 | 2410226 | 97.1 | 2.9 | 73.6 |
| Day2-113 | 3554313 | 90.0 | 10.0 | 36.9 |
| Day2-114 | 2462448 | 93.2 | 6.8 | 57.0 |
| Day2-115 | 2807757 | 93.8 | 6.2 | 58.5 |
| Day2-116 | 1547509 | 88.4 | 11.6 | 33.1 |
| Day2-117 | 2365408 | 92.5 | 7.5 | 57.3 |
| Day2-118 | 2096486 | 95.5 | 4.5 | 68.8 |
| Day3-1 | 2909734 | 86.7 | 13.3 | 17.2 |
| Day3-2 | 3293855 | 85.3 | 14.7 | 8.6 |
| Day3-3 | 3796373 | 86.5 | 13.5 | 10.6 |
| Day3-4 | 3014799 | 86.2 | 13.8 | 10.7 |
| Day3-5 | 3208761 | 86.0 | 14.0 | 10.2 |
| Day3-6 | 3205203 | 84.3 | 15.7 | 6.3 |
| Day3-7 | 3612156 | 86.0 | 14.0 | 11.7 |
| Day3-8 | 2538581 | 83.1 | 16.9 | 7.1 |
| Day3-9 | 3097036 | 84.3 | 15.7 | 7.0 |
| Day3-10 | 3109515 | 85.2 | 14.8 | 7.5 |
| Day3-11 | 3657519 | 85.4 | 14.6 | 6.8 |
| Day3-12 | 3598460 | 85.6 | 14.4 | 9.4 |
| Day3-13 | 3857296 | 86.8 | 13.2 | 11.4 |
| Day3-14 | 4380009 | 87.2 | 12.8 | 13.8 |
| Day3-15 | 3508476 | 86.4 | 13.6 | 22.1 |
| Day3-16 | 3007040 | 85.4 | 14.6 | 5.6 |
| Day3-17 | 3538040 | 86.3 | 13.7 | 7.0 |
| Day3-18 | 3471819 | 85.7 | 14.3 | 12.2 |
| Day3-19 | 3418594 | 85.3 | 14.7 | 6.5 |
| Day3-20 | 2776169 | 85.5 | 14.5 | 4.6 |
| Day3-21 | 3386860 | 83.9 | 16.1 | 5.6 |
| Day4-1 | 2512088 | 91.0 | 9.0 | 3.3 |
| Day4-2 | 237349 | 88.9 | 11.1 | 2.9 |
| Day4-3 | 2209597 | 88.7 | 11.3 | 3.4 |
| Day4-4 | 2545036 | 90.1 | 9.9 | 3.7 |
| Day4-5 | 2789708 | 89.2 | 10.8 | 6.6 |
| Day4-6 | 3333524 | 92.0 | 8.0 | 8.1 |
| Day4-7 | 2147804 | 88.6 | 11.4 | 3.4 |
| Day4-8 | 3954984 | 91.7 | 8.3 | 8.0 |
| Day4-9 | 3435188 | 90.4 | 9.6 | 7.3 |
| Day4-10 | 4439136 | 87.4 | 12.6 | 8.5 |
| Day4-11 | 4718270 | 91.8 | 8.2 | 21.0 |
| Day4-12 | 3295232 | 88.3 | 11.7 | 3.1 |
| Day4-13 | 2643263 | 91.1 | 8.9 | 10.4 |
| Day4-14 | 5160664 | 93.0 | 7.0 | 14.6 |
| Day4-15 | 4396294 | 89.4 | 10.6 | 6.4 |
| Day4-16 | 5604739 | 94.7 | 5.3 | 31.7 |
| Day4-17 | 4488570 | 89.6 | 10.4 | 6.1 |
| Day4-18 | 4428250 | 91.1 | 8.9 | 8.4 |
| Day4-19 | 5191296 | 91.8 | 8.2 | 7.3 |
| Day4-20 | 4619674 | 90.3 | 9.7 | 7.7 |
| Day4-21 | 5334058 | 89.3 | 10.7 | 6.9 |
| Day4-22 | 3144079 | 90.5 | 9.5 | 6.8 |
| Day4-23 | 3114956 | 88.7 | 11.3 | 6.1 |
| Day4-24 | 6467589 | 91.3 | 8.7 | 12.8 |
| Day4-25 | 3606348 | 87.6 | 12.4 | 4.6 |
| Day4-26 | 5399174 | 92.3 | 7.7 | 12.7 |
| Day4-27 | 3666688 | 89.6 | 10.4 | 3.0 |
| Day4-28 | 4298976 | 91.5 | 8.5 | 4.9 |
| Day4-29 | 5083878 | 92.2 | 7.8 | 8.5 |
| Day4-30 | 4751914 | 90.5 | 9.5 | 7.4 |
| Day4-31 | 3486362 | 90.0 | 10.0 | 3.6 |
| Day4-32 | 2844116 | 91.7 | 8.3 | 18.9 |
| Day4-33 | 2330343 | 88.1 | 11.9 | 2.6 |
| Day4-34 | 2844014 | 87.3 | 12.7 | 3.6 |
| Day4-35 | 1966120 | 87.1 | 12.9 | 3.2 |
| Day4-36 | 2911691 | 88.8 | 11.2 | 5.4 |
| Day4-37 | 3332447 | 90.9 | 9.1 | 8.0 |
| Day4-38 | 2464554 | 90.0 | 10.0 | 5.6 |
| Day4-39 | 4709950 | 90.9 | 9.1 | 13.5 |
| Day4-40 | 4138274 | 91.9 | 8.1 | 10.4 |
| Day4-41 | 3810021 | 88.4 | 11.6 | 2.3 |
| Day4-42 | 5103061 | 91.6 | 8.4 | 13.8 |
| Day4-43 | 3888809 | 87.5 | 12.5 | 7.0 |
| Day4-44 | 4751964 | 87.7 | 12.3 | 9.3 |
| Day4-45 | 4208059 | 91.6 | 8.4 | 9.0 |
| Day4-46 | 4389228 | 89.1 | 10.9 | 4.9 |
| Day4-47 | 4912179 | 84.6 | 15.4 | 17.9 |
| Day4-48 | 5897013 | 89.3 | 10.7 | 5.3 |
| Day4-49 | 4126511 | 89.1 | 10.9 | 3.8 |
| Day4-50 | 5843328 | 89.6 | 10.4 | 6.6 |
| Day4-51 | 5676481 | 89.0 | 11.0 | 28.4 |
| Day4-52 | 6070802 | 90.1 | 9.9 | 23.7 |
| Day4-53 | 3475745 | 88.7 | 11.3 | 3.3 |
| Day4-54 | 5887451 | 90.1 | 9.9 | 6.9 |
| Day4-55 | 5439110 | 90.0 | 10.0 | 5.2 |
| Day4-56 | 5152065 | 87.2 | 12.8 | 6.5 |
| Day4-57 | 5982702 | 91.5 | 8.5 | 23.1 |
| Day4-58 | 4466965 | 91.4 | 8.6 | 4.8 |
| Day4-59 | 4062662 | 89.9 | 10.1 | 3.3 |
| Day4-60 | 3645661 | 89.5 | 10.5 | 5.9 |
| Day4-61 | 215556 | 87.3 | 12.7 | 12.0 |
| Day4-62 | 4150903 | 88.5 | 11.5 | 4.4 |
| Day4-63 | 3821974 | 91.4 | 8.6 | 8.6 |
| Day4-64 | 5572853 | 87.3 | 12.7 | 7.5 |
| Day4-65 | 4981137 | 89.4 | 10.6 | 12.4 |
| Day4-66 | 6195393 | 91.4 | 8.6 | 12.5 |
| Day4-67 | 4421955 | 87.5 | 12.5 | 16.3 |
| Day4-68 | 3521532 | 87.6 | 12.4 | 4.6 |
| Day4-69 | 1064893 | 90.5 | 9.5 | 12.1 |
| Day4-70 | 1404139 | 86.7 | 13.3 | 5.0 |
| Day4-71 | 5390391 | 90.9 | 9.1 | 6.5 |
| Day4-72 | 4102383 | 88.0 | 12.0 | 7.1 |
| Day4-73 | 5280794 | 89.2 | 10.8 | 8.5 |
| Day4-74 | 4056904 | 89.0 | 11.0 | 11.7 |
| Day4-75 | 4372793 | 90.5 | 9.5 | 14.5 |
| Day4-76 | 5086293 | 87.4 | 12.6 | 8.9 |
| Day4-77 | 5387463 | 86.5 | 13.5 | 9.7 |
| Day4-78 | 3705902 | 88.1 | 11.9 | 4.9 |
| Day4-79 | 3906320 | 87.1 | 12.9 | 5.5 |
| Day4-80 | 3334828 | 88.2 | 11.8 | 18.9 |
| Day4-81 | 4543410 | 88.5 | 11.5 | 4.8 |
| Day4-82 | 4838562 | 92.2 | 7.8 | 25.7 |
| Day4-83 | 5524961 | 89.4 | 10.6 | 6.1 |
| Day4-84 | 3219732 | 87.1 | 12.9 | 6.9 |
| Day4-85 | 5791874 | 90.4 | 9.6 | 14.2 |
| Day4-86 | 3093063 | 86.6 | 13.4 | 4.3 |
| Day4-87 | 4297457 | 89.3 | 10.7 | 13.0 |
| Day4-88 | 3755072 | 88.9 | 11.1 | 4.8 |
| Day4-89 | 2865061 | 89.1 | 10.9 | 5.4 |
| Day4-90 | 4510234 | 92.5 | 7.5 | 20.3 |
| Day4-91 | 6164704 | 89.5 | 10.5 | 12.0 |
| Day4-92 | 2674293 | 87.6 | 12.4 | 2.9 |
| Day4-93 | 3662908 | 87.4 | 12.6 | 5.9 |
| Day4-94 | 2731931 | 88.5 | 11.5 | 6.5 |
| Day4-95 | 2373159 | 87.3 | 12.7 | 3.1 |
| Day4-96 | 2492055 | 89.1 | 10.9 | 4.2 |
| Day4-97 | 2640683 | 89.1 | 10.9 | 8.6 |
| Day4-98 | 2482042 | 88.9 | 11.1 | 3.5 |
| Day4-99 | 2514843 | 87.0 | 13.0 | 3.9 |
| Day4-100 | 3271422 | 87.2 | 12.8 | 3.0 |
| Day4-101 | 3770702 | 88.4 | 11.6 | 5.4 |
| Day4-102 | 5057897 | 89.7 | 10.3 | 11.9 |
| Day4-103 | 2871117 | 86.7 | 13.3 | 3.3 |
| Day4-104 | 4383179 | 89.3 | 10.7 | 14.3 |
| Day4-105 | 3486803 | 88.6 | 11.4 | 5.4 |
| Day4-106 | 3992782 | 87.6 | 12.4 | 5.7 |
| Day4-107 | 3950722 | 90.3 | 9.7 | 9.4 |
| Day4-108 | 5153840 | 93.7 | 6.3 | 32.0 |
| Day4-109 | 2922007 | 88.6 | 11.4 | 3.5 |
| Day4-110 | 5623250 | 92.1 | 7.9 | 13.5 |
| Day4-111 | 4644992 | 92.0 | 8.0 | 22.3 |
| Day4-112 | 3853437 | 87.9 | 12.1 | 7.2 |
| Day4-113 | 2293675 | 85.1 | 14.9 | 6.5 |
| Day4-114 | 3674858 | 93.1 | 6.9 | 48.1 |
| Day4-115 | 6050217 | 87.3 | 12.7 | 9.4 |
| Day4-116 | 4057988 | 88.4 | 11.6 | 7.5 |
| Day4-117 | 4399563 | 88.5 | 11.5 | 8.5 |
| Day4-118 | 5867785 | 90.9 | 9.1 | 14.4 |
| Day4-119 | 5695465 | 88.8 | 11.2 | 9.5 |
| Day4-120 | 5025104 | 89.0 | 11.0 | 8.6 |
| Day4-121 | 5470248 | 88.6 | 11.4 | 8.7 |
| Day4-122 | 668514 | 88.9 | 11.1 | 10.1 |
| Day4-123 | 3476134 | 85.9 | 14.1 | 4.1 |
| Day4-124 | 3264661 | 86.8 | 13.2 | 3.6 |
| Day4-125 | 5372286 | 86.8 | 13.2 | 10.4 |
| Day4-126 | 4909627 | 86.7 | 13.3 | 7.7 |
| Day4-127 | 2714678 | 90.4 | 9.6 | 24.6 |
| Day4-128 | 4351069 | 86.6 | 13.4 | 4.3 |
| Day4-129 | 5522563 | 86.9 | 13.1 | 7.3 |

| Capturing efficiency *P. chabaudi* infection | | |  |  |
| --- | --- | --- | --- | --- |
|  | Day 2 | Day 3 | Day 4 | Total (%) |
| 1 cell | 139 | 73 | 143 | 75% |
| 0 cells | 49 | 16 | 35 | 20% |
| 2+ cells | 4 | 7 | 14 | 5% |

**Supplementary Table 8**

**Traditional academic point system:**

- .90-1 = excellent (A)
- .80-.90 = good (B)
- .70-.80 = fair (C)
- .60-.70 = poor (D)
- .50-.60 = fail (F)
